# Supplementary material for: Waveform distortion for temperature compensation and synchronization in circadian rhythms: An approach based on the renormalization group method
Source: PLoS Comput Biol. 2025 Jul 22;21(7):e1013246. doi: 10.1371/journal.pcbi.1013246 (PMC12282898; doi:10.1371/journal.pcbi.1013246)
Supplement: S1 Text — (PDF) [file pcbi.1013246.s001.pdf]

Supporting Information for

Waveform distortion for temperature compensation and synchronization in circadian rhythms: An approach based on the renormalization group method

Shingo Gibo[1,4*], Teiji Kunihiro[2], Tetsuo Hatsuda[1,3], and Gen Kurosawa[1*]

[1]RIKEN Center for Interdisciplinary Theoretical and Mathematical Sciences (iTHEMS), Japan

[2]Yukawa Institute for Theoretical Physics (YITP), Kyoto University, Japan

[3]Kavli Institute for the Physics and Mathematics of the Universe, WPI, University of Tokyo, Kashiwa, Japan

[4]Current Address: Biomedical Mathematics Group, Pioneer Research Center for Mathematical and Computational Sciences, Institute for Basic Science, Republic of Korea

[*]Correspondence: shingogibo@ibs.re.kr (S.G), g.kurosawa@riken.jp (G.K)

This PDF includes:

Supplementary Text S1 to S5

Supplementary Figures S1 to S7

Supplementary Tables S1 to S2

Supplementary Text

S.1. Brief introduction of the renormalization group (RG) method using a simple model with Hopf bifurcation

In this section, we introduce the RG method [47] in a geometrical manner as formulated in [48, 52, 53] with a simpler prescription without the redundant 'time-splitting' procedure. In this aim, we use a generic model with a Hopf bifurcation. A more detailed account of the method is given in [53].

Let us consider the model equation

$$Lx(t) = F(x(t); \varepsilon) \tag{28}$$

where $x(t)$ is a state variable of our dynamics, $L = \sum_{n=1}^{N} a_n (d/dt)^n$ is a linear differential operator, $F(x(t); \varepsilon)$ is a nonlinear function of $x(t)$, and ε is an internal parameter of F, which acts as a bifurcation parameter of the system. We assume that the model has a fixed point x_0 satisfying the equation $F(x_0; 0) = 0$, which is destabilized for $\varepsilon > 0$ through the Hopf bifurcation. We are interested in the derivation of the reduced equation and an approximate but valid solution in a global domain of time around the critical point of the Hopf bifurcation. Thus, we apply the perturbation theory and express the solution around an arbitrary time $t = t_0$ belonging to a global domain in the asymptotic regime (see below) in a power series of ε as follows:

$$x(t; t_0) = x_0 + \varepsilon u_1(t; t_0) + \varepsilon^2 u_2(t; t_0) + \varepsilon^3 u_3(t; t_0) + o(\varepsilon^3). \tag{29}$$

Substituting Eq. (29) into Eq. (28) and equating the terms with the same powers of ε, we obtained

$$O(\varepsilon^1) : L' u_1 = 0, \tag{30}$$

$$O(\varepsilon^2) : L' u_2 = f_1(u_1), \tag{31}$$

$$O(\varepsilon^3) : L' u_3 = f_2(u_1, u_2), \tag{32}$$

where

$$L' = L - (\partial F / \partial x)|_{x=x_0, \varepsilon=0},$$

and $f_1(u_1)$ and $f_2(u_1, u_2)$ are nonlinear functions that depend on $F(x_0)$. Because Hopf bifurcation occurs at $\varepsilon = 0$, two of the eigenvalues of the linear differential operator L' are written as $\pm i\omega_0$, with ω_0 being a real number, and the others have negative real parts as $Re(\lambda_k) < 0$ $(k = 1, ..., N-2)$.

Then, the first-order solution can be expressed as

$$u_1(t;t_0) = A(t_0)\cos(\omega_0 t + \theta(t_0)) + \sum_{k=1}^{N-2} c_k(t_0)e^{\lambda_k t}, \tag{33}$$

where $A(t_0)$, $\theta(t_0)$, and $c_k(t_0)$ are the integral constants which are assumed to depend on the initial time t_0.

Next, we consider the asymptotic regime as $t \to \infty$ so that the second term describing the transient behavior has virtually become negligible. Then, the first-order solution in this asymptotic regime can be expressed only by the first term as

$$u_1(t \to \infty; t_0) = A(t_0)\cos(\omega_0 t + \theta(t_0)). \tag{34}$$

Next, we proceed to the second-order equation. Substituting Eq. (34) into Eq. (31), we have

$$L' u_2 = b_1 A\cos(\omega_0 t + \theta) + b_2 A^2 \cos(2(\omega_0 t + \theta)) + b_2 A^2, \tag{35}$$

where b_k $(k = 1, 2)$ are constants depending on $f_1(u_1)$. It is to be noted that the inhomogeneous part (r.h.s.) contains a term proportional to $\cos(\omega_0 t + \theta)$, which is a zero mode of the linear operator that gives rise to secular terms in the particular solutions of the inhomogeneous equation. The general solution to Eq. (35) is given as a sum of a particular solution to the inhomogeneous equation and the general solution to the homogeneous equation. Now, it is possible and convenient to choose the coefficients of the latter so that all of the secular terms vanish at $t = t_0$ [48], which leads to the second-order solution as

$$\begin{aligned} u_2(t;t_0) =& (t - t_0) d_1 A\cos(\omega_0 t + \theta) + (t - t_0) d_2 A \sin(\omega_0 t + \theta) \\ &+ d_3 A^2 \cos(2(\omega_0 t + \theta)) + d_4 A^2 \sin(2(\omega_0 t + \theta)) + d_5 A^2, \end{aligned} \tag{36}$$

where d_k $(k = 1 \cdots 5)$ are constants depending on the right-hand side of Eq. (35).

Similarly, the third-order solution takes the form of

$$\begin{aligned}u_3(t;t_0) =&(t-t_0)(f_{1a}A^3+f_{1b}A)\cos(\omega_0 t+\theta)+(t-t_0)(f_{2a}A^3+f_{2b}A)\sin(\omega_0 t+\theta)\\&+(t-t_0)^2 f_3 A\cos(\omega_0 t+\theta)+(t-t_0)^2 f_4 A\sin(\omega_0 t+\theta)+f_5 A^2\cos(2(\omega_0 t+\theta))\\&+f_6 A^2\sin(2(\omega_0 t+\theta))+(t-t_0)f_7 A^2\cos(2(\omega_0 t+\theta))+(t-t_0)f_8 A^2\sin(2(\omega_0 t+\theta))\\&+f_9 A^3\cos(3(\omega_0 t+\theta))+f_{10}A^3\sin(3(\omega_0 t+\theta))+f_{11}A^2+(t-t_0)f_{12}A^2,\end{aligned}\tag{37}$$

where f_{1a}, f_{1b}, f_{2a}, f_{2b}, and f_k $(k=3\cdots 12)$ are constants depending on Eqs. (32), (34), and (36). Note that the solution is constructed so that the secular terms vanish at $t=t_0$.

Thus, collecting all of the terms, the approximate solution to Eq. (28) up to the third order of ε reads

$$\begin{aligned}x(t;t_0) =&x_0+\varepsilon A\cos(\omega_0 t+\theta)+\varepsilon^2\{(t-t_0)d_1 A\cos(\omega_0 t+\theta)+(t-t_0)d_2 A\sin(\omega_0 t+\theta)\\&+d_3 A^2\cos(2(\omega_0 t+\theta))+d_4 A^2\sin(2(\omega_0 t+\theta))+d_5 A^2\}\\&+\varepsilon^3\{(t-t_0)(f_{1a}A^3+f_{1b}A)\cos(\omega_0 t+\theta)+(t-t_0)(f_{2a}A^3+f_{2b}A)\sin(\omega_0 t+\theta)\\&+(t-t_0)^2 f_3 A\cos(\omega_0 t+\theta)+(t-t_0)^2 f_4 A\sin(\omega_0 t+\theta)+f_5 A^2\cos(2(\omega_0 t+\theta))\\&+f_6 A^2\sin(2(\omega_0 t+\theta))+(t-t_0)f_7 A^2\cos(2(\omega_0 t+\theta))+(t-t_0)f_8 A^2\sin(2(\omega_0 t+\theta))\\&+f_9 A^3\cos(3(\omega_0 t+\theta))+f_{10}A^3\sin(3(\omega_0 t+\theta))+f_{11}A^2+(t-t_0)f_{12}A^2\}+o(\varepsilon^3).\end{aligned}\tag{38}$$

Because Eq. (38) contains the secular terms, this solution is valid only locally around $t=t_0$, but it exhibits a divergent behavior as $|t-t_0|$ goes infinity. In fact, this is a rather common behavior occurring in naïve perturbation expansions.

Next, we use a geometrical viewpoint to circumvent the disastrous situation following [48]. The solution (38) gives a family of curves with t_0 being the parameter specifying each curve in the t-x plane. Each curve gives a good approximate solution to the original equation in a local domain around $t=t_0$. The idea is that the *envelope curve* of the family of curves hopefully gives an approximate but valid solution in the global domain including the arbitrary time t_0. Indeed, this has rigorously been demonstrated to be the case [48, 49, 52]. Now, the envelope curve can be constructed using the following envelope equation [48]:

$$\left.\frac{dx(t;t_0)}{dt_0}\right|_{t_0=t}=\left.\frac{\partial x}{\partial t_0}\right|_{t_0=t}+\frac{dA(t_0)}{dt_0}\left.\frac{\partial x}{\partial A}\right|_{t_0=t}+\frac{d\theta(t_0)}{dt_0}\left.\frac{\partial x}{\partial \theta}\right|_{t_0=t}=0.\tag{39}$$

Note that we have taken into account the fact that the integral constants A and θ depend on the 'initial time' $t=t_0$, and (39) actually gives the dynamical equations for these variables. As will be done shortly, the

insertion of the solutions to the dynamic equation into (38) gives an approximate but globally valid solution to the original equation. Because the envelope equation (39) takes a similar form as the RG equation in quantum field theory, it is also called the RG equation, and the asymptotic/global analysis based on this equation was named the RG method [80].

Substituting Eq. (38) into Eq. (39), we have

$$\begin{aligned}
0 =&\varepsilon \left\{ \frac{dA}{dt} - \varepsilon^2 f_{1a} A^3 - \varepsilon(d_1 + \varepsilon f_{1b})A \right\} \cos(\omega_0 t + \theta) \\
&+ \varepsilon A \left\{ -\frac{d\theta}{dt} - \varepsilon^2 f_{2a} A^2 - \varepsilon(d_2 + \varepsilon f_{2b}) \right\} \sin(\omega_0 t + \theta) \\
&+ \varepsilon^2 A \left\{ 2(d_3 + \varepsilon f_5)\frac{dA}{dt} + 2(d_4 + \varepsilon f_6) A \frac{d\theta}{dt} - \varepsilon f_7 A \right\} \cos(2(\omega_0 t + \theta)) \\
&+ \varepsilon^2 A \left\{ 2(d_4 + \varepsilon f_6)\frac{dA}{dt} - 2(d_3 + \varepsilon f_5) A \frac{d\theta}{dt} - \varepsilon f_8 A \right\} \sin(2(\omega_0 t + \theta)) \\
&+ 3\varepsilon^3 A^2 \left\{ f_9 \frac{dA}{dt} + f_{10} A \frac{d\theta}{dt} \right\} \cos(3(\omega_0 t + \theta)) + 3\varepsilon^3 A^2 \left\{ f_{10} \frac{dA}{dt} - f_9 A \frac{d\theta}{dt} \right\} \sin(3(\omega_0 t + \theta)) \\
&+ 2\varepsilon^2 (d_5 + \varepsilon f_{11}) A \frac{dA}{dt} - \varepsilon^3 f_{12} A + o(\varepsilon^3).
\end{aligned} \tag{40}$$

Because dA/dt and $d\theta/dt$ are of order ε, the coefficients $\cos(2(\omega_0 t + \theta))$, $\sin(2(\omega_0 + \theta))$, $\cos(3(\omega_0 t + \theta))$, $\sin(3(\omega_0 t + \theta))$, and $A dA/dt$ are of order ε^3 or higher. To make Eq. (40) hold for any t, we only must ensure that the coefficients of the independent functions, namely $\cos(\omega_0 t + \theta)$ and $\sin(\omega_0 t + \theta)$, vanish, and hence, we have

$$\frac{dA}{dt} = \varepsilon^2 f_{1a} A^3 + \varepsilon(d_1 + \varepsilon f_{1b})A + o(\varepsilon^2), \tag{41}$$

$$\frac{d\theta}{dt} = -\varepsilon^2 f_{2a} A^2 - \varepsilon(d_2 + \varepsilon f_{2b}) + o(\varepsilon^2), \tag{42}$$

which are the dynamic equations governing the 'integral constants' A and θ. We now see that the integral constants have been lifted to dynamic variables through the RG/envelope equation. The amplitude equation (41) can be readily solved analytically. For instance, when $f_{1a} < 0$ and $d_1 + \varepsilon f_{1b} > 0$, it yields

$$A(t) = A_0 \frac{\mathcal{A}}{\sqrt{\mathcal{A}^2 + (A_0^2 - \mathcal{A}^2)e^{-2\alpha t}}}, \tag{43}$$

where $\alpha = \varepsilon(d_1 + \varepsilon f_{1b})$ and

$$A_0 = \sqrt{-\frac{d_1 + \varepsilon f_{1b}}{\varepsilon f_{1a}}}, \tag{44}$$

with $\mathcal{A}$ being the initial amplitude. Equation (43) indicates that the amplitude approaches A_0 monotonically as $t \to \infty$, implying that A_0 is nothing but the amplitude of the limit cycle admitted in the original equation (28). Furthermore, Eq. (42) indicates that the angular frequency on the limit cycle reads

$$\omega = \omega_0 + (d\theta/dt)|_{A=A_0} = \omega_0 - \varepsilon \frac{d_2 f_{1a} - d_1 f_{2a}}{f_{1a}}, \tag{45}$$

which is constant. The globally valid solution is given as the envelope of the family of curves, as previously stated. Thus, the solution on the limit cycle, which valid in a global domain in the asymptotic regime, reads

$$\begin{aligned} x(t) =& x(t;t_0)|_{t_0=t} \\ =& x_0 + \varepsilon A_0 \cos(\omega t + \theta_0) + \varepsilon^2\{d_3 A_0^2 \cos(2(\omega t + \theta_0)) + d_4 A_0^2 \sin(2(\omega t + \theta_0)) + d_5 A_0^2\} \\ &+ \varepsilon^3\{f_5 A_0^2 \cos(2(\omega t + \theta_0)) + f_6 A_0^2 \sin(2(\omega t + \theta_0)) + f_9 A_0^3 \cos(3(\omega t + \theta_0)) \\ &+ f_{10} A_0^3 \sin(3(\omega t + \theta_0)) + f_{11} A_0^2\} + o(\varepsilon^3). \end{aligned} \tag{46}$$

The RG method is a powerful method for obtaining a globally valid solution. This method can be applied to various models including discrete, stochastic, and partial differential equations, as given in [53, 58].

References

80. Goldenfeld N, Martin O, Oono Y. Intermediate asymptotics and renormalization group theory. J Sci Comput. 1989;4: 355-372.
