# Supplementary material for: Waveform distortion for temperature compensation and synchronization in circadian rhythms: An approach based on the renormalization group method
Source: PLoS Comput Biol. 2025 Jul 22;21(7):e1013246. doi: 10.1371/journal.pcbi.1013246 (PMC12282898; doi:10.1371/journal.pcbi.1013246)
Supplement: S2 Text — (PDF) [file pcbi.1013246.s002.pdf]

S.2 Derivation of a time-evolution solution in a circadian rhythm model using the RG method

In this subsection, we applied the RG method to derive an approximate but globally valid solution of the circadian clock model given by Eq. (2)-(4), which is a system of first-order equations with three variables. We first convert the system into a single equation with higher-order derivatives as

$$\frac{d^3x_3}{dt^3} + s_1\frac{d^2x_3}{dt^2} + s_2\frac{dx_3}{dt} + s_3x_3 = p_1p_2f(x_3) \tag{47}$$

where $s_1 = k_1 + k_2 + k_3$, $s_2 = k_1k_2 + k_2k_3 + k_3k_1$, $s_3 = k_1k_2k_3$. To obtain the approximate solution, we set the transcriptional regulator $f(x_3)$ as r/x_3^n. This model has a fixed point

$$x_0 = \left(\frac{s_3}{p_1p_2r}\right)^{-\frac{1}{n+1}}, \tag{48}$$

which is destabilized through Hopf bifurcation for

$$n > n_0 \equiv \frac{s_4}{s_3}, \tag{49}$$

with $s_4 = (k_1 + k_2)(k_2 + k_3)(k_3 + k_1)$.

Then, we expand the solution around $t = t_0$ as a series of ε

$$x_3(t;t_0) = x_0 + \varepsilon u_1(t;t_0) + \varepsilon^2u_2(t;t_0) + \varepsilon^3u_3(t;t_0) + O(\varepsilon^3). \tag{50}$$

Substituting Eq. (50) into Eq. (47) and equating the coefficients with the same powers of ε, we obtain

$$O(\varepsilon) : \frac{d^3u_1}{dt^3} + s_1\frac{d^2u_1}{dt^2} + s_2\frac{du_1}{dt} + s_1s_2u_1 = 0, \tag{51}$$

$$O(\varepsilon^2) : \frac{d^3u_2}{dt^3} + s_1\frac{d^2u_2}{dt^2} + s_2\frac{du_2}{dt} + s_1s_2u_2 = -s_3u_1 + B_1u_1^2, \tag{52}$$

$$O(\varepsilon^3) : \frac{d^3u_3}{dt^3} + s_1\frac{d^2u_3}{dt^2} + s_2\frac{du_3}{dt} + s_1s_2u_3 = -s_3u_2 + B_2u_1u_2 + B_3u_1^2 + B_4u_1^3, \tag{53}$$

where

$$B_1 = \frac{s_4 s_2 s_1}{2 s_3} \left(\frac{s_3}{p_1 p_2 r} \right)^{\frac{s_3}{s_1 s_2}}, \tag{54}$$

$$B_2 = \frac{s_4 s_2 s_1}{s_3} \left(\frac{s_3}{p_1 p_2 r} \right)^{\frac{s_3}{s_1 s_2}}, \tag{55}$$

$$B_3 = \left\{ s_4 + \frac{s_3}{2} - \left(\frac{s_4 s_3}{2 s_2 s_1} \ln \left(\frac{s_3}{p_1 p_2 r} \right) \right) \right\} \left(\frac{s_3}{p_1 p_2 r} \right)^{\frac{s_3}{s_1 s_2}}, \tag{56}$$

$$B_4 = \frac{(s_2 s_1 + s_3) s_4 s_1 s_2}{6 s_3^2} \left(\frac{2 s_3}{p_1 p_2 r} \right)^{\frac{s_3}{s_1 s_2}}. \tag{57}$$

Because Eq. (51) has three eigenvalues, namely $\lambda_{1,2} = \pm i\sqrt{s_2}$ and $\lambda_3 = -s_1$, the general solution of Eq. (51) reads

$$u_1(t; t_0) = A(t_0) \cos(\omega_0 t + \theta(t_0)) + c(t_0) e^{-s_1 t}, \qquad (\omega_0 := \sqrt{s_2}), \tag{58}$$

where A, θ, and c are integral constants that depend on initial time t_0.

Considering the asymptotic regime in which the second term in Eq. (58) is so small and negligible, then the first-order solution can be written as

$$u_1(t; t_0) = A(t_0) \cos(\omega_0 t + \theta(t_0)). \tag{59}$$

Substituting Eq. (59) into Eq. (52), we obtain

$$\frac{d^3 u_2}{dt^3} + s_1 \frac{d^2 u_2}{dt^2} + s_2 \frac{du_2}{dt} + s_1 s_2 u_2 = -s_3 A \cos(\omega_0 t + \theta) + CA^2 \cos(2\omega_0 t + 2\theta) + CA^2, \tag{60}$$

where

$$C = \frac{s_4 s_1 s_2}{4 s_3} \left(\frac{s_3}{p_1 p_2 r} \right)^{\frac{s_3}{s_1 s_2}}. \tag{61}$$

Equation (60) is an inhomogeneous equation that contains a zero mode of the linear operator, and the

solution having a suitable form for applying the RG method is written as

$$
\begin{aligned}
u_2(t;t_0) =& D_1 A(t-t_0)\cos(\omega_0 t+\theta) - D_2 A(t-t_0)\sin(\omega_0 t+\theta) \\
& - D_3 A^2\cos(2\omega_0 t+2\theta) - D_4 A^2 \sin(2\omega_0 t+2\theta) + D_5 A^2,
\end{aligned} \tag{62}
$$

where the coefficients are

$$D_1 = \frac{s_3}{2(s_1^2+s_2)}, \tag{63}$$

$$D_2 = \frac{s_1 s_3}{2\sqrt{s_2}(s_1^2+s_2)}, \tag{64}$$

$$D_3 = \frac{s_4 s_1^2}{12(s_1^2+4s_2)s_3}\left(\frac{s_3}{p_1 p_2 r}\right)^{\frac{s_3}{s_1 s_2}}, \tag{65}$$

$$D_4 = \frac{s_4 s_1 \sqrt{s_2}}{6(s_1^2+4s_2)s_3}\left(\frac{s_3}{p_1 p_2 r}\right)^{\frac{s_3}{s_1 s_2}}, \tag{66}$$

$$D_5 = \frac{s_4}{4s_3}\left(\frac{s_3}{p_1 p_2 r}\right)^{\frac{s_3}{s_1 s_2}}. \tag{67}$$

Tentatively, after collecting all the obtained terms, we have the approximate solution in the second order as

$$
\begin{aligned}
x_3(t;t_0) =& x_0 + \varepsilon A\cos(\omega_0 t+\theta) + \varepsilon^2\{D_1 A(t-t_0)\cos(\omega_0 t+\theta) - D_2 A(t-t_0)\sin(\omega_0 t+\theta) \\
& - D_3 A^2\cos(2\omega_0 t+2\theta) - D_4 A^2\sin(2\omega_0 t+2\theta) + D_5 A^2\} + o(\varepsilon^2).
\end{aligned} \tag{68}
$$

Because it contains the secular terms, the solution diverges as $|t-t_0|$ goes infinity. To resum the would-be divergent terms, we apply the RG equation $dx_3(t,t_0)/dt_0|_{t_0=t}$, which leads to the equations governing the amplitude and phase as

$$dA/dt = \varepsilon s_3 A/2(s_1^2+s_2),$$

and

$$d\theta/dt = \varepsilon s_1 s_3/2\sqrt{s_2}(s_1^2+s_2),$$

nicely describing the slow motions of the amplitude and phase, respectively. However, it fails to describe a transitional behavior approaching a limit cycle, as indicated by the present model.

Therefore, we analyzed the third-order equation, which might lead to a limit cycle solution. Substituting Eqs. (59) and (62) into Eq. (53), we have

$$\frac{d^3u_3}{dt^3} + s_1\frac{d^2u_3}{dt^2} + s_2\frac{du_3}{dt} + s_1s_2u_3 = E_1A^3\cos(\omega_0t+\theta) - E_2A^3\sin(\omega_0t+\theta)$$
$$- E_3A(t-t_0)\cos(\omega_0t+\theta) + E_4A(t-t_0)\sin(\omega_0t+\theta) + E_5A^2\cos(2\omega_0t+2\theta)$$
$$+ E_6A^2\sin(2\omega_0t+2\theta) + E_7A^2(t-t_0)\cos(2\omega_0t+2\theta) - E_8A^2(t-t_0)\sin(2\omega_0t+2\theta)$$
$$- E_9A^3\cos(3\omega_0t+3\theta) - E_{10}A^3\sin(3\omega_0t+3\theta) + E_{11}A^2 + E_{12}A^2(t-t_0). \tag{69}$$

where

$$E_1 = \frac{(s_1^2s_2 - 4s_1^2s_3 + 6s_1s_2^2 - 18s_2s_3)s_4s_1s_2}{12(s_1^2+4s_2)s_3^2}\left(\frac{s_3}{p_1p_2r}\right)^{\frac{2s_3}{s_1s_2}}, \tag{70}$$

$$E_2 = \frac{s_4^2s_1^2\sqrt{s_2^3}}{12(s_1^2+4s_2)s_3^2}\left(\frac{s_3}{p_1p_2r}\right)^{\frac{2s_3}{s_1s_2}}, \tag{71}$$

$$E_3 = \frac{s_3^2}{2(s_1^2+s_2)}, \tag{72}$$

$$E_4 = \frac{s_1s_3^2}{2(s_1^2+s_2)\sqrt{s_2}}, \tag{73}$$

$$E_5 = \left\{\frac{7s_1^3s_2 - 4s_1^2s_3 + 24s_1s_2^2 - 12s_2s_3}{12(s_1^2+4s_2)} - \frac{s_4s_3}{4s_1s_2}\ln\left(\frac{s_3}{p_1p_2r}\right)\right\}\left(\frac{s_3}{p_1p_2r}\right)^{\frac{s_3}{s_1s_2}}, \tag{74}$$

$$E_6 = \frac{s_4s_1\sqrt{s_2}}{6(s_1^2+4s_2)}\left(\frac{s_3}{p_1p_2r}\right)^{\frac{s_3}{s_1s_2}}, \tag{75}$$

$$E_7 = \frac{s_4s_1s_2}{4(s_1^2+s_2)}\left(\frac{s_3}{p_1p_2r}\right)^{\frac{s_3}{s_1s_2}}, \tag{76}$$

$$E_8 = \frac{s_4s_1^2\sqrt{s_2}}{4(s_1^2+s_2)}\left(\frac{s_3}{p_1p_2r}\right)^{\frac{s_3}{s_1s_2}}, \tag{77}$$

$$E_9 = \frac{((s_1^2+2s_2)s_1 + 2s_3)s_4s_1s_2^2}{12(s_1^2+4s_2)s_3^2}\left(\frac{s_3}{p_1p_2r}\right)^{\frac{2s_3}{s_1s_2}}, \tag{78}$$

$$E_{10} = \frac{s_4^2s_1^2\sqrt{s_2^3}}{12(s_1^2+4s_2)s_3^2}\left(\frac{s_3}{p_1p_2r}\right)^{\frac{2s_3}{s_1s_2}}, \tag{79}$$

$$E_{11} = \frac{1}{4}\left\{s_1s_2 - \frac{s_4s_3}{s_1s_2}\ln\left(\frac{s_3}{p_1p_2r}\right)\right\}\left(\frac{s_3}{p_1p_2r}\right)^{\frac{2s_3}{s_1s_2}}, \tag{80}$$

$$E_{12} = \frac{s_4s_1s_2}{4(s_1^2+s_2)}\left(\frac{s_3}{p_1p_2r}\right)^{\frac{s_3}{s_1s_2}}. \tag{81}$$

The solution to Eq. (69) is given by

$$
\begin{aligned}
u_3(t;t_0) = & -(F_{1a}A^3 + F_{1b}A)(t-t_0)\cos(\omega_0 t+\theta) + (F_{2a}A^3 + F_{2b}A)(t-t_0)\sin(\omega_0+\theta) \\
& - F_3 A(t-t_0)^2\cos(\omega_0 t+\theta) - F_4 A(t-t_0)^2\sin(\omega_0 t+\theta) + F_5 A^2\cos(2\omega_0 t+2\theta) \\
& + F_6 A^2\sin(2\omega_0 t+2\theta) - F_7 A^2(t-t_0)\cos(2\omega_0 t+2\theta) \\
& + F_8 A^2(t-t_0)\sin(2\omega_0 t+2\theta) + F_9 A^3\cos(3\omega_0 t+3\theta) + F_{10}A^3\sin(3\omega_0 t+3\theta) \\
& + F_{11}A^2 + F_{12}A^2(t-t_0)
\end{aligned} \tag{82}
$$

where

$$F_{1a} = \frac{(2s_1 s_2^2 - (s_1^2 + 6s_2)s_3)s_4 s_1 s_2}{8(s_1^2 + 4s_2)(s_1^2 + s_2)s_3^2} \left(\frac{s_3}{p_1 p_2 r}\right)^{\frac{2s_3}{s_1 s_2}}, \tag{83}$$

$$F_{1b} = \frac{s_1 s_3^2}{(s_1^2 + s_2)^3}, \tag{84}$$

$$F_{2a} = \frac{((s_1^2 + 7s_2)s_1 s_2 - (4s_1^2 + 19s_2)s_3)s_4 s_1^2 \sqrt{s_2}}{24(s_1^2 + s_2)(s_1^2 + 4s_2)s_3^2} \left(\frac{s_3}{p_1 p_2 r}\right)^{\frac{2s_3}{s_1 s_2}}, \tag{85}$$

$$F_{2b} = \frac{((s_1^2 + 6s_2)s_1^2 - 3s_2^2)s_3^2}{8(s_1^2 + s_2)^3 \sqrt{s_2^3}}, \tag{86}$$

$$F_3 = \frac{(s_1^2 - s_2)s_3^2}{8(s_1^2 + s_2)s_2}, \tag{87}$$

$$F_4 = \frac{s_1 s_3^2}{4(s_1^2 + s_2)\sqrt{s_2}}, \tag{88}$$

$$F_5 = \left\{ \frac{s_4 s_3}{12(s_1^2 + 4s_2)s_2^2} \ln\left(\frac{s_3}{p_1 p_2 r}\right) - \frac{(3s_1^5 + 4s_1^3 s_2 + 11 s_1^2 s_3 + 64 s_1 s_2^2 - 52 s_2 s_3)s_1}{36(s_1^2 + s_2)(s_1^2 + 4s_2)^2} \right\} \left(\frac{s_3}{p_1 p_2 r}\right)^{\frac{s_3}{s_1 s_2}}, \tag{89}$$

$$F_6 = \left\{ \frac{s_4 s_3}{6(s_1^2 + 4s_2)s_1 \sqrt{s_2^3}} \ln\left(\frac{s_3}{p_1 p_2 r}\right) - \frac{7s_1^5 s_2 - s_1^4 s_3 - 8s_1^3 s_2^2 + 38 s_1^2 s_2 s_3 + 48 s_1 s_2^3 - 24 s_2^2 s_3}{36(s_1^2 + s_2)(s_1^2 + 4s_2)^2 \sqrt{s_2}} \right\} \left(\frac{s_3}{p_1 p_2 r}\right)^{\frac{s_3}{s_1 s_2}}, \tag{90}$$

$$F_7 = \frac{s_4 s_1^2}{4(s_1^2 + s_2)(s_1^2 + 4s_2)} \left(\frac{s_3}{p_1 p_2 r}\right)^{\frac{s_3}{s_1 s_2}}, \tag{91}$$

$$F_8 = \frac{(s_1^2 - 2s_2)s_4 s_1}{12(s_1^2 + s_2)(s_1^2 + 4s_2)\sqrt{s_2}} \left(\frac{s_3}{p_1 p_2 r}\right)^{\frac{s_3}{s_1 s_2}}, \tag{92}$$

$$F_9 = \frac{(s_1(s_1^2 - s_2) + 5s_3)s_4 s_1^2 s_2}{96(s_1^2 + 4s_2)(s_1^2 + 9s_2)s_3^2} \left(\frac{s_3}{p_1 p_2 r}\right)^{\frac{2s_3}{s_1 s_2}}, \tag{93}$$

$$F_{10} = \frac{(2(2s_1^2 + 3s_2)s_1 s_2 + (6s_2 - s_1^2)s_3)s_4 s_1 \sqrt{s_2}}{96(s_1^2 + 4s_2)(s_1^2 + 9s_2)s_3^2} \left(\frac{s_3}{p_1 p_2 r}\right)^{\frac{2s_3}{s_1 s_2}}, \tag{94}$$

$$F_{11} = \left\{ \frac{(s_1^3 + s_3)}{4(s_1^2 + s_2)s_1} - \frac{s_4 s_3}{4s_1^2 s_2^2} \ln\left(\frac{s_3}{p_1 p_2 r}\right) \right\} \left(\frac{s_3}{p_1 p_2 r}\right)^{\frac{s_3}{s_1 s_2}}, \tag{95}$$

$$F_{12} = \frac{s_4}{4(s_1^2 + s_2)} \left(\frac{s_3}{p_1 p_2 r}\right)^{\frac{s_3}{s_1 s_2}}. \tag{96}$$

To obtain a globally valid solution using Eq. (82), we apply the RG method, which utilizes the RG equa-

tion

$$\begin{aligned}\left.\frac{dx_3(t;t_0)}{dt_0}\right|_{t_0=t} &= \left.\frac{\partial x_3(t;t_0)}{\partial t_0}\right|_{t_0=t} + \left.\frac{dA}{dt_0}\frac{\partial x_3(t;t_0)}{\partial A}\right|_{t_0=t} + \left.\frac{d\theta}{dt_0}\frac{\partial x_3(t;t_0)}{\partial \theta}\right|_{t_0=t} \\ &= \left\{\varepsilon\frac{dA}{dt} - \varepsilon D_1 A + \varepsilon^3(F_{1a}A^3 + F_{1b}A)\right\}\cos(\omega_0 t + \theta) \\ &\quad + \left\{-\varepsilon A\frac{d\theta}{dt} + \varepsilon^2 D_2 A - \varepsilon^3(F_{2a}A^3 + F_{2b}A)\right\}\sin(\omega_0 t + \theta) = 0, \end{aligned} \tag{97}$$

where we have neglected the higher-order terms $o(\varepsilon^3)$. For Eq. (97) to hold for any t, the coefficients of the two independent functions should vanish. Thus, we obtain the dynamic equations for A and θ:

$$\frac{dA}{dt} = \varepsilon D_1 A - \varepsilon^2(F_{1a}A^3 + F_{1b}A) + o(\varepsilon^2), \tag{98}$$

$$\frac{d\theta}{dt} = \varepsilon D_2 - \varepsilon^2(F_{2a}A^2 + F_{2b}) + o(\varepsilon^2). \tag{99}$$

The amplitude equation (98) has a new fixed point

$$A_0 = \sqrt{\frac{D_1 - \varepsilon F_{1b}}{\varepsilon F_{1a}}} = \sqrt{\frac{4((s_1^2+s_2)^2 - 2\varepsilon s_1 s_3)(s_1^2+4s_2)s_3^3}{\varepsilon(2s_1s_2^2 - (s_1^2+6s_2)s_3)(s_1^2+s_2)^2 s_4 s_1 s_2}\left(\frac{p_1p_2r}{s_3}\right)^{\frac{s_3}{s_1s_2}}}, \tag{100}$$

which is nothing but the amplitude of the desired limit cycle. The phase function $\theta(t)$ on the limit cycle is expressed as

$$\theta(t) = (\varepsilon D_2 - \varepsilon^2(F_{2a}A_0^2 + F_{2b}) + o(\varepsilon^2))t + \theta_0, \tag{101}$$

where θ_0 is the integral constant and it gives the initial phase at $t = 0$. Substituting Eqs. (64), (85), (86) and (100) into (101), $\theta(t)$ is reduced to

$$\theta(t) = \left\{-\varepsilon\frac{s_1s_3s_4}{6(2s_1s_2^2 - (s_1^2+6s_2)s_3)\sqrt{s_2}} + o(\varepsilon)\right\}t + \theta_0. \tag{102}$$

Thus, the solution describing the limit cycle, which is valid in a global domain in the asymptotic regime, is given by

$$\begin{aligned} x_3(t) =& x_3(t;t_0)|_{t_0=t} \\ =& x_0 + \varepsilon A_0\cos(\omega t + \theta_0) \\ &- \varepsilon^2\{D_3A_0^2\cos(2(\omega t+\theta_0)) + D_4A_0^2\sin(2(\omega t+\theta_0))\} + o(\varepsilon^2). \end{aligned} \tag{103}$$

In particular, if the initial phase is set to be $\theta_0 = -\pi/2$, we have Eq. (13).
