# Supplementary material for: Waveform distortion for temperature compensation and synchronization in circadian rhythms: An approach based on the renormalization group method
Source: PLoS Comput Biol. 2025 Jul 22;21(7):e1013246. doi: 10.1371/journal.pcbi.1013246 (PMC12282898; doi:10.1371/journal.pcbi.1013246)
Supplement: S3 Text — (PDF) [file pcbi.1013246.s003.pdf]

S.3 Numerical analyses of Relógio model

In this paper, we numerically and analytically showed that the waveform is more distorted at higher temperatures when the period is stable to changes in temperature, using the Goodwin model. Additionally, we have numerically showed that the same conclusion can be obtained in a realistic mammalian circadian clock model [21, 32, 70]. In this section, we analyze another mammalian circadian clock model, called Relógio model [73], to examine the generality of our conclusion.

This model includes 19 variables: 5 mRNAs (*Per, Cry, Rev-erb, Ror*, and *Bmal*), 3 nuclear proteins (REV-ERB, ROR, and BMAL), 6 cytoplasmic proteins (PER, phosphorylated PER, CRY, REV-ERB, ROR, and BMAL), 2 nuclear protein complexes (PER/CRY and phosphorylated PER/CRY), and 3 cytoplasmic protein complexes (CLOCK/BMAL, PER/CRY and phosphorylated PER/CRY). The model mainly consists of two feedback loops, the ROR/*Bmal*/REV-ERB loop and the PER/CRY loop, generating robust oscillations. We first generated 30 reference parameter sets, with each parameter ranging from one-half and to double the original values, except for 11 Hill coefficients of transcription and 5 exogenous mRNA levels. Next, the reaction parameters were multiplied by a random factor within the range of 1.5-2.5 (20 sets for each reference parameter set), except for 11 activation/inhibition rates, 5 transcription fold activations, 11 Hill coefficients of transcription, and 5 exogenous mRNA levels because their units are dimensionless or arbitrary. We quantified the relative period (= period at fast reaction/period at slow reaction) and the relative NS (= NS at fast reaction/NS at slow reaction) for all 19 variables. The change in NS depends on the variable being measured. The NS of cytoplasmic PER/CRY becomes larger when the period is stable or increases with higher reaction rates (S5A Fig.), whereas this tendency is less apparent for *Cry* mRNA (S5B Fig.). Notably, the geometric mean of NS for all variables clearly increases when the period is stable or increases with higher reaction rates (S5C Fig.). This suggests that waveform distortion plays an important role in period stability across various circadian clock models.
