# Supplementary material for: Waveform distortion for temperature compensation and synchronization in circadian rhythms: An approach based on the renormalization group method
Source: PLoS Comput Biol. 2025 Jul 22;21(7):e1013246. doi: 10.1371/journal.pcbi.1013246 (PMC12282898; doi:10.1371/journal.pcbi.1013246)
Supplement: S4 Text — (PDF) [file pcbi.1013246.s004.pdf]

S.4 Numerical and RG analyses of the Lotka-Volterra model

Equation (5) and the numerical simulation indicate that non-sinusoidal power (NS) tends to be larger when the period hardly changes and is stable in response to increases of the parameter values specifying the degradation rates, as presented in Fig. 1B. This suggests that the waveform becomes more distorted at higher temperatures when the circadian period is temperature-compensated. It was previously reported that the same conclusion holds for other oscillatory models, including a realistic mammalian circadian clock model, a post-translational model in cyanobacteria, and the van der Pol oscillator [32, 70]. However, it is important to note that the findings using specific mathematical models might not be universally applicable to other models and actual organisms. Therefore, one should examine whether the waveform also plays a crucial role in the stability of the period in other oscillatory models.

Thus, we conduct a numerical simulation to test the possible period-waveform correlation in the Lotka-Volterra model as done for the circadian clock model. Needless to say, the Lotka-Volterra model is one of the most extensively studied mathematical models in biology [74], and it effectively explains population dynamics in prey-predator systems. The Lotka-Volterra model is given as a system with two variables as

$$\frac{dx}{dt} = ax - \varepsilon xy, \tag{104}$$

$$\frac{dy}{dt} = -by + \varepsilon' xy, \tag{105}$$

where $x(t)$ and $y(t)$ are numbers of prey and predators. Parameter a is the growth rate of prey, b is the death rate of predators, ε is the death rate of prey attributable to predation, and ε' is the growth rate of predators (S6A Fig.).

In the present numerical simulation, we first generated 100 reference parameter sets corresponding to the reference temperature. The values of the model parameters a and b were generated randomly with a uniform distribution between 0 and 1, and similarly, the values of the other model parameters ε and ε' were also randomly assigned values between 0 and 0.5. The period obtained for each parameter set with the initial condition $(x_0, y_0) = (2, 2)$ was denoted as τ_1. Next, a, b, ε, and ε' were multiplied by a random factor within the range of 1.1-1.9 to simulate the increase in temperature, yielding 49 oscillatory parameter sets. Each resulting new period was denoted as τ_2, and thus, the ratio of the period $= \tau_2/\tau_1$, which is called the relative period, was obtained.

The numerical simulations reveal a consistent positive correlation between NS and the relative period (S6BC Fig.). A notable point is that the value of NS tends to increase along with the period when larger multi-

plicative factors are used in the simulation. This result again suggests that our findings that the waveform becomes more distorted when the period remains relatively stable in response to increased parameter values is a rather universal phenomenon not restricted to the behavior observed in the circadian clock model (Fig. 2A).

The period of the Lotka-Volterra model, together with its approximate but globally valid solution, was previously derived analytically by one of the present authors [49, 52] on the basis of the RG method; see also the pioneering work [81] based on a different method. Next, we will demonstrate that the expression explicitly reveals that the period of the Lotka-Volterra model almost linearly increases with the waveform distortion for the average of the prey-predator time series (i.e. $\overline{NS}$). In the mathematical analysis, it proved convenient to use new variables $(\xi(t), \eta(t))$ defined as

$$x(t) = (b + \varepsilon\xi(t))/\varepsilon', \quad y(t) = a/\varepsilon + \eta(t).$$

The RG method performed in [50, 52] in the second order of ε leads to

$$\begin{aligned}\xi(t) = &\left(1 - \varepsilon^2 \frac{a-b}{4ab^2}\frac{A^2}{12}\right) A \sin\Theta - \varepsilon^2 \frac{1}{b\sqrt{ab}}\frac{A^2}{24}\cos\Theta - \varepsilon\frac{1}{\sqrt{ab}}\frac{A^2}{6}\sin(2\Theta) \\ &- \varepsilon\frac{1}{b}\frac{A^2}{3}\cos(2\Theta) - \varepsilon^2\frac{3a-b}{4ab^2}\frac{A^3}{8}\sin(3\Theta) + \varepsilon^2\frac{1}{b\sqrt{ab}}\frac{A^2}{8}\cos(3\Theta) + o(\varepsilon^2), \qquad (106)\\ \eta(t) = &\varepsilon^2\frac{1}{b^2}\frac{A^3}{24}\sin\Theta - \frac{\sqrt{ab}}{b}\left(1 + \varepsilon^2\frac{a-b}{4ab^2}\frac{A^2}{12}\right)A\cos\Theta - \varepsilon\frac{\sqrt{ab}}{b^2}\frac{A^2}{6}\sin(2\Theta) \\ &+ \varepsilon\frac{1}{b}\frac{A^2}{3}\cos(2\Theta) + \varepsilon^2\frac{1}{b^2}\frac{A^3}{8}\sin(3\Theta) + \varepsilon^2\frac{a-3b}{4b^2\sqrt{ab}}\frac{A^3}{8}\cos(3\Theta) + o(\varepsilon^2), \qquad (107)\end{aligned}$$

where A and θ are the integral constants, which are to be determined by the initial condition, and $\Theta = \tilde{\omega}t + \theta$, with $\tilde{\omega}$ being the angular frequency given by

$$\tilde{\omega} = \sqrt{ab}\left\{1 - \frac{\varepsilon^2 A^2(a+b)}{24ab^2}\right\}, \qquad (108)$$

from which we have the formula of the period of the system after some manipulation as

$$\tau = \frac{2\pi}{\tilde{\omega}} = \frac{2\pi}{\sqrt{ab}}\left\{\frac{2}{5}\left(1 + \frac{5}{2}\frac{\varepsilon^2 A^2(a+b)}{24ab^2}\right) + \frac{3}{5}\right\} + o(\varepsilon^2). \qquad (109)$$

From the waveforms for $\xi(t)$ and $\eta(t)$ given by (106) and (107), respectively, we can obtain the waveform

distortion of each variable as follows:

$$NS^{(\xi)} = 1 + \frac{\varepsilon^2 A^2 (4a + b)}{24ab^2} + o(\varepsilon^2), \tag{110}$$

$$NS^{(\eta)} = 1 + \frac{\varepsilon^2 A^2 (a + 4b)}{24ab^2} + o(\varepsilon^2). \tag{111}$$

It is notable that the mean of $NS^{(\xi)}$ and $NS^{(\eta)}$ takes the form

$$\overline{NS} = \frac{1}{2}(NS^{(\xi)} + NS^{(\eta)}) = 1 + \frac{5}{2}\frac{\varepsilon^2 A^2 (a + b)}{24ab^2} + o(\varepsilon^2). \tag{112}$$

Indeed, comparing (112) and (109), we arrive at

$$\tau = \frac{2\pi}{\sqrt{ab}}\left(\frac{2}{5}\overline{NS} + \frac{3}{5}\right) + o(\varepsilon^2), \tag{113}$$

which states that the period and the mean waveform distortion, namely $\overline{NS}$, are linearly dependent on each other, and they tend to increase (or decrease) in a parallel manner. This is what we aimed to demonstrate for the Lotka-Volterra model.

References

81. Frame JS. Explicit solutions in two species Volterra systems. J Theor Biol. 1974;43: 73-81.
