# Supplementary material for: Waveform distortion for temperature compensation and synchronization in circadian rhythms: An approach based on the renormalization group method
Source: PLoS Comput Biol. 2025 Jul 22;21(7):e1013246. doi: 10.1371/journal.pcbi.1013246 (PMC12282898; doi:10.1371/journal.pcbi.1013246)
Supplement: S5 Text — (PDF) [file pcbi.1013246.s005.pdf]

S.5 Numerical and RG analyses of the van der Pol model

We consider the van del Pol model as follows:

$$\frac{d^2x}{dt^2} + x = \varepsilon(1 - x^2)\frac{dx}{dt}, \tag{114}$$

which is known as one of the fundamental non-linear oscillator models. Previously, two of the authors derived the period formula $\tau = 2\pi[\sum_{j=1}^{\infty} |a_j|^2 j^2 / \sum_{j=1}^{\infty} |a_j|^2]^{1/2}$, meaning that the period of the model is also proportional to NS [32]. Then, we derive the approximate solution of the model using the RG method and confirm the proportionality between the period and the waveform distortion NS in detail. First, we represent the local solution around $t = t_0$ as a perturbation series

$$x(t; t_0) = x_0(t; t_0) + \varepsilon x_1(t; t_0) + \varepsilon^2 x_2(t; t_0) + o(\varepsilon^2). \tag{115}$$

Then, substituting Eq. (115) into Eq. (114) and equating the terms with the same powers of ε, we obtain

$$O(\varepsilon^0) : \frac{d^2x_0}{dt^2} + x_0 = 0, \tag{116}$$

$$O(\varepsilon^1) : \frac{d^2x_1}{dt^2} + x_1 = (1 - x_0^2)\frac{dx_0}{dt}, \tag{117}$$

$$O(\varepsilon^2) : \frac{d^2x_2}{dt^2} + x_2 = (1 - x_0^2)\frac{dx_1}{dt} - 2x_0x_1\frac{dx_0}{dt}. \tag{118}$$

The solution for the zeroth-order equation (116) is

$$x_0(t; t_0) = A(t_0)\cos(t + \theta(t_0)), \tag{119}$$

where A and θ are integral constants and they potentially depend on initial time t_0. Then, substituting Eq. (119) into Eq. (117), we have

$$\frac{d^2x_1}{dt^2} + x_1 = -A\left(1 - \frac{A^2}{4}\right)\sin(t + \theta) + \frac{A^3}{4}\sin(3t + 3\theta). \tag{120}$$

By solving the solution of Eq. (120) around $t = t_0$, the first-order solution is given by

$$x_1(t;t_0) = \frac{A}{2}\left(1-\frac{A^2}{4}\right)(t-t_0)\cos(t+\theta) - \frac{A^3}{32}\sin(3t+3\theta). \tag{121}$$

Similarly, by substituting zero-th and first-order solutions (119) and (121) into Eq. (118), the second-order equation is

$$\begin{aligned}\frac{d^2x_2}{dt^2}+x_2 =&F_1(A)\cos(t+\theta) - F_2(A)(t-t_0)\sin(t+\theta) + F_3(A)\cos(3t+3\theta)\\ &- F_4(A)(t-t_0)\sin(3t+3\theta) + F_5(A)\cos(5t+5\theta),\end{aligned} \tag{122}$$

where

$$F_1(A) = \frac{A}{2}\left(\frac{13}{64}A^4 - A^2 + 1\right), \tag{123}$$

$$F_2(A) = \frac{A}{2}\left(\frac{3}{16}A^4 - A^2 + 1\right), \tag{124}$$

$$F_3(A) = \frac{A^3}{32}\left(\frac{5}{2}A^2 - 7\right), \tag{125}$$

$$F_4(A) = \frac{3A^3}{8}\left(\frac{1}{4}A^2 - 1\right), \tag{126}$$

$$F_5(A) = \frac{5A^5}{128}. \tag{127}$$

The solution of the second-order equation (122) is

$$\begin{aligned}x_2(t;t_0) =&\frac{1}{4}(2F_1(A) - F_2(A))(t-t_0)\sin(t+\theta) + \frac{1}{4}F_2(A)(t-t_0)^2\cos(t+\theta)\\ &+ \frac{1}{32}(-4F_3(A)+3F_4(A))\cos(3t+3\theta) + \frac{1}{8}F_4(A)(t-t_0)\sin(3t+3\theta)\\ &- \frac{1}{24}F_5(A)\cos(5t+5\theta).\end{aligned} \tag{128}$$

Therefore, the perturbative solution up to the second-order of ε is

$$\begin{aligned}x(t;t_0) =&A\cos(t+\theta) + \varepsilon\left\{\frac{A}{2}\left(1-\frac{A^2}{4}\right)(t-t_0)\cos(t+\theta) - \frac{A^3}{32}\sin(3t+3\theta)\right\}\\ &+ \varepsilon^2\left\{\frac{1}{4}(2F_1(A) - F_2(A))(t-t_0)\sin(t+\theta) + \frac{1}{4}F_2(A)(t-t_0)^2\cos(t+\theta)\right.\\ &+ \frac{1}{32}(-4F_3(A)+3F_4(A))\cos(3t+3\theta) + \frac{1}{8}F_4(A)(t-t_0)\sin(3t+3\theta)\\ &\left.-\frac{1}{24}F_5(A)\cos(5t+5\theta).\right\} + o(\varepsilon^2).\end{aligned} \tag{129}$$

To obtain the globally valid solution using Eq. (129), we apply the RG method, which utilizes the RG

equation

$$\left.\frac{dx}{dt_0}\right|_{t_0=t} = \left\{\frac{dA}{dt} + \varepsilon\frac{1}{2}A\left(\frac{1}{4}A^2 - 1\right)\right\}\cos(t+\theta) + \left\{-A\frac{d\theta}{dt} - \varepsilon^2\frac{1}{4}(2F_1(A) - F_2(A))\right\}\sin(t+\theta) = 0, \tag{130}$$

where we have neglected the higher-order terms $o(\varepsilon^2)$. For Eq. (130) to hold for any t, the coefficients of the two independent functions should vanish. Thus, we end up with the dynamical equations for A and θ

$$\frac{dA}{dt} = \frac{1}{2}\varepsilon A\left(1 - \frac{1}{4}A^2\right) \tag{131}$$

$$\frac{d\theta}{dt} = -\varepsilon^2\frac{1}{4A}(2F_1(A) - F_2(A)) = -\varepsilon^2\frac{1}{8}\left(\frac{7}{32}A^4 - A^2 + 1\right) \tag{132}$$

Equation (131) has two fixed points, namely $A = 0$ and 2. The amplitude A asymptotically approaches $A = 2$, which is the limit cycle. Therefore, the dynamical behavior of $\theta(t)$ on the limit cycle reads

$$\theta(t) = -\varepsilon^2\frac{1}{16}t + \theta_0, \tag{133}$$

where θ_0 is the initial phase at $t = 0$. Thus, the globally valid solution on the limit cycle is given by

$$x(t) = 2\cos(\omega t + \theta_0) - \varepsilon\frac{1}{4}\sin(3\omega t + 3\theta_0) - \varepsilon^2\frac{3}{32}\cos(3\omega t + 3\theta_0) - \varepsilon^2\frac{5}{96}\cos(5\omega t + 5\theta_0) + o(\varepsilon^2), \tag{134}$$

where the angular frequency ω up to the second order is expressed as

$$\omega = 1 - \varepsilon^2\frac{1}{16} + o(\varepsilon^2). \tag{135}$$

Using Eqs. (134) and (135), the period and NS of the van der Pol model read

$$\tau = \frac{2\pi}{\omega} = \frac{2\pi}{1 - \varepsilon^2/16 + o(\varepsilon^2)} = 2\pi\left(1 + \varepsilon^2\frac{1}{16} + o(\varepsilon^2)\right), \tag{136}$$

$$NS = \left[\frac{\sum_{j=1}^{\infty}|a_j|^2 j^2}{\sum_{j=1}^{\infty}|a_j|^2}\right]^{\frac{1}{2}} = \left[\frac{1 + \varepsilon^2(9/64) + o(\varepsilon^2)}{1 + \varepsilon^2/64 + o(\varepsilon^2)}\right]^{\frac{1}{2}} = 1 + \varepsilon^2\frac{1}{16} + o(\varepsilon^2), \tag{137}$$

Accordingly,

$$\tau = 2\pi NS. \tag{138}$$

This expression clearly illustrates that the period τ and the waveform distortion NS in the van der Pol model tend to increase (or decrease) together in a proportional manner, which was also demonstrated using signal processing methods (S7 Fig.) [32].
