# Supplementary material for: Waveform distortion for temperature compensation and synchronization in circadian rhythms: An approach based on the renormalization group method
Source: PLoS Comput Biol. 2025 Jul 22;21(7):e1013246. doi: 10.1371/journal.pcbi.1013246 (PMC12282898; doi:10.1371/journal.pcbi.1013246)
Supplement: S1 Fig — (PDF) [file pcbi.1013246.s006.pdf]

Supplementary Figures

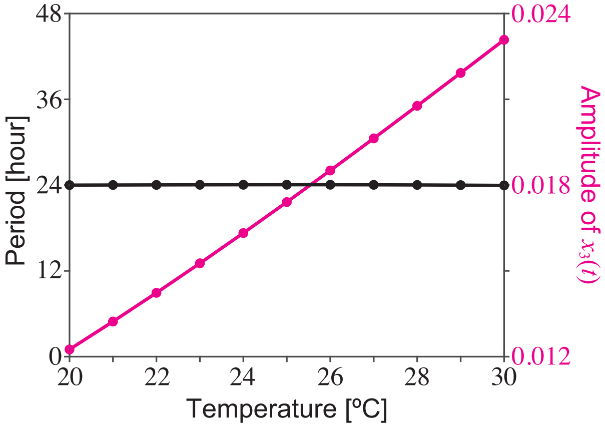

Figure S 1: The period and amplitude of Goodwin model as function of temperature.
