# Supplementary material for: Waveform distortion for temperature compensation and synchronization in circadian rhythms: An approach based on the renormalization group method
Source: PLoS Comput Biol. 2025 Jul 22;21(7):e1013246. doi: 10.1371/journal.pcbi.1013246 (PMC12282898; doi:10.1371/journal.pcbi.1013246)
Supplement: S2 Fig — We first generated 100 reference parameter sets yielding oscillations. Then the reaction rates of k1, k2, k3, p1, p2, and r were increased individually by 1%. We show the elasticity (=∂lnA1/∂lnqi, where qi represents k1, k2, k3, p1, p2, and r). The bar indicates the average, and the line indicates the standard deviation. The right column shows the summation of the elasticities (=∑∂lnA1/∂lnqi). (PDF) [file pcbi.1013246.s007.pdf]

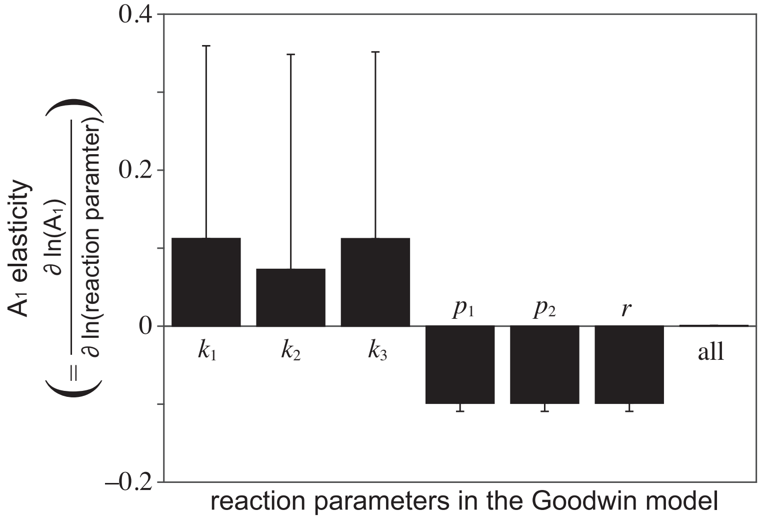

Figure S 2: Sensitivity of A_1 to the reaction rates in the Goodwin model. We first generated 100 reference parameter sets yielding oscillations. Then the reaction rates of k_1, k_2, k_3, p_1, p_2, and r were increased individually by 1%. We show the elasticity ($= \partial \ln A_1 / \partial \ln q_i$, where q_i represents k_1, k_2, k_3, p_1, p_2, and r). The bar indicates the average, and the line indicates the standard deviation. The right column shows the summation of the elasticities ($= \sum \partial \ln A_1 / \partial \ln q_i$).
