# Supplementary material for: Waveform distortion for temperature compensation and synchronization in circadian rhythms: An approach based on the renormalization group method
Source: PLoS Comput Biol. 2025 Jul 22;21(7):e1013246. doi: 10.1371/journal.pcbi.1013246 (PMC12282898; doi:10.1371/journal.pcbi.1013246)
Supplement: S3 Fig — (A) Re-plot of Fig 4B in [59]. The average curves of tim-luc at 18 and 29°C are extracted using WebPlotDigitizer at 1-h intervals. Noise uniformly distributed between –0.4 and 0.4 is added to generate 100 time series datasets. Spline interpolation is applied to set the sampling interval to 0.1 h. The interpolated time series data at 18 (cyan) and 29 °C (magenta) are plotted. (B) Distribution of the quantified waveform distortion (NS) from the data at 18 (cyan) and 29 °C (magenta). The noisy time series data are detrended by multiplying an exponential function to align local maxima at 18 °C or local minima at 29 °C. The Fourier coefficients of the detrended time-series are quantified using GHA. NS values were estimated from the coefficients up to the third harmonics. Dots represent the NS values for each data set, and horizontal lines represent their average values. The estimated NS values increased significantly at higher temperatures (t = 14.7971, df = 191, p < 0.001). (PDF) [file pcbi.1013246.s008.pdf]

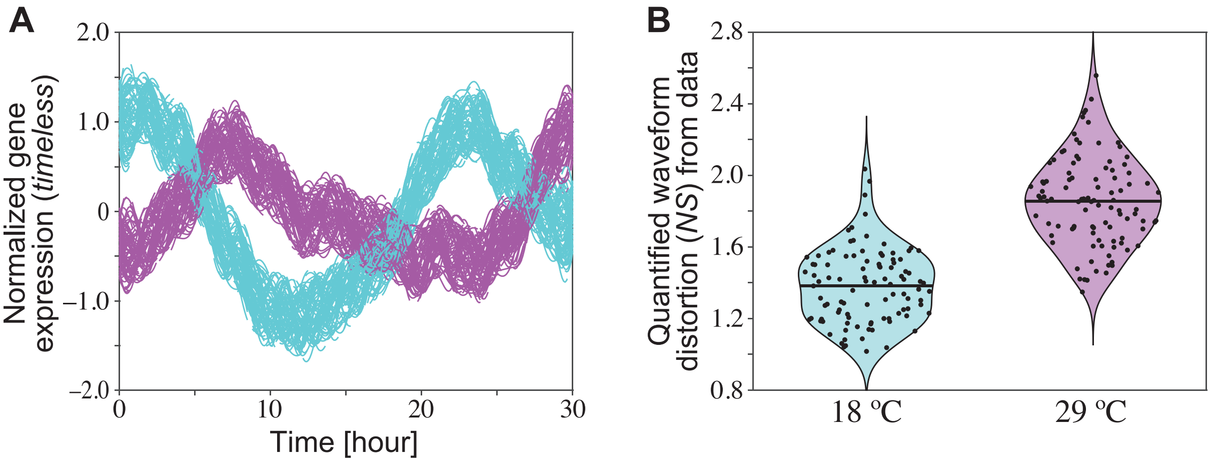


Figure S 3: Analysis of the circadian waveform of the Drosophila mutant perL at different temperatures using previously reported experimental data [59]. (A) Re-plot of Fig. 4B in [59]. The average curves of *tim-luc* at 18 and 29°C are extracted using WebPlotDigitizer at 1-h intervals. Noise uniformly distributed between −0.4 and 0.4 is added to generate 100 time series datasets. Spline interpolation is applied to set the sampling interval to 0.1 h. The interpolated time series data at 18 (cyan) and 29 °C (magenta) are plotted. (B) Distribution of the quantified waveform distortion ($NS$) from the data at 18 (cyan) and 29 °C (magenta). The noisy time series data are detrended by multiplying an exponential function to align local maxima at 18 °C or local minima at 29 °C. The Fourier coefficients of the detrended time-series are quantified using GHA. $NS$ values are estimated from the coefficients up to the third harmonics. Dots represent the $NS$ values for each data set, and horizontal lines represent their average values. The estimated $NS$ values increased significantly at higher temperatures ($t$ = 14.7971, $df$ = 191, $p < 0.001$).
