# Supplementary material for: Waveform distortion for temperature compensation and synchronization in circadian rhythms: An approach based on the renormalization group method
Source: PLoS Comput Biol. 2025 Jul 22;21(7):e1013246. doi: 10.1371/journal.pcbi.1013246 (PMC12282898; doi:10.1371/journal.pcbi.1013246)
Supplement: S4 Fig — (A) Re-plot of Fig. 5C in [21]. The curves of Per2-luc between 5 and 30 hours at 30 and 37°C are extracted using WebPlotDigitizer at 1-h intervals. The amplitude is normalized so that the maximum value is one. Noise uniformly distributed between –0.05 and 0.05 is added to generate 100 time series datasets. Spline interpolation is applied to set the sampling interval to 0.1 h. The interpolated time series data at 30 (cyan) and 37 °C (magenta) are plotted. (B) Distribution of the quantified waveform distortion (NS) from the data at 30 (cyan) and 37 °C (magenta). The noisy time series data are detrended by multiplying an exponential function to align local maxima at 30 °C or local minima at 37 °C. The Fourier coefficients of the detrended time-series are quantified using GHA. NS values are estimated from the coefficients up to the third harmonics using Eq (1) with m = 4 and q = 2. Dots represent the NS values for each data set, and horizontal lines represent their average values. The estimated NS values increased significantly at higher temperatures (t = 2.9793, df = 189, p = 0.0016). (PDF) [file pcbi.1013246.s009.pdf]

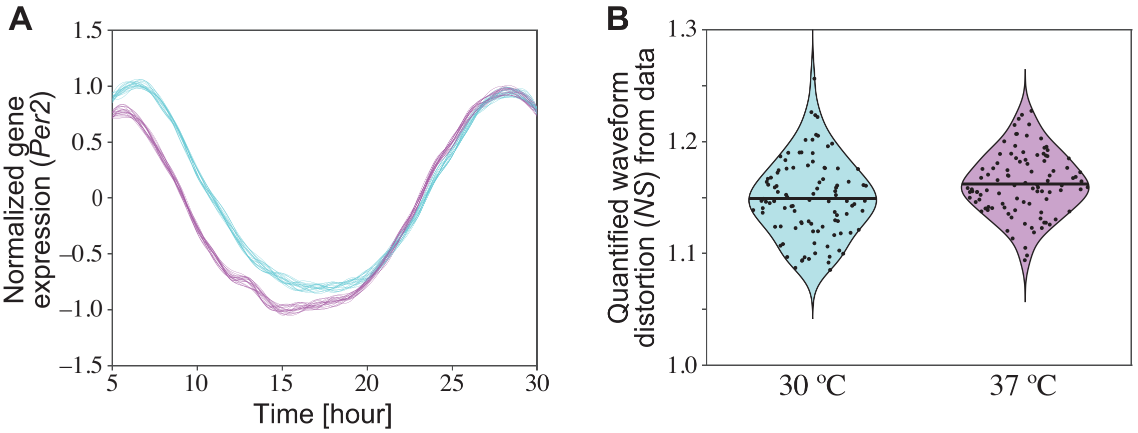

Figure S 4: Analysis of the circadian waveform of *Per2-luc* in mice at different temperatures using previously reported experimental data [21]. (A) Re-plot of Fig. 5C in [21]. The curves of *Per2-luc* between 5 and 30 hours at 30 and 37°C are extracted using WebPlotDigitizer at 1-h intervals. The amplitude is normalized so that the maximum value is one. Noise uniformly distributed between -0.05 and 0.05 is added to generate 100 time series datasets. Spline interpolation is applied to set the sampling interval to 0.1 h. The interpolated time series data at 30 (cyan) and 37 °C (magenta) are plotted. (B) Distribution of the quantified waveform distortion (NS) from the data at 30 (cyan) and 37 °C (magenta). The noisy time series data are detrended by multiplying an exponential function to align local maxima at 30 °C or local minima at 37 °C. The Fourier coefficients of the detrended time-series are quantified using GHA. NS values are estimated from the coefficients up to the third harmonics using Eq. (1) with $m = 4$ and $q = 2$. Dots represent the NS values for each data set, and horizontal lines represent their average values. The estimated NS values increased significantly at higher temperatures ($t = 2.9793$, $df = 189$, $p = 0.0016$).
