# Supplementary material for: Waveform distortion for temperature compensation and synchronization in circadian rhythms: An approach based on the renormalization group method
Source: PLoS Comput Biol. 2025 Jul 22;21(7):e1013246. doi: 10.1371/journal.pcbi.1013246 (PMC12282898; doi:10.1371/journal.pcbi.1013246)
Supplement: S5 Fig — We plot relative NS of cytoplasmic PER/CRY complex (A), Cry mRNA (B), and geometric mean of NS for all variables (C). (PDF) [file pcbi.1013246.s010.pdf]

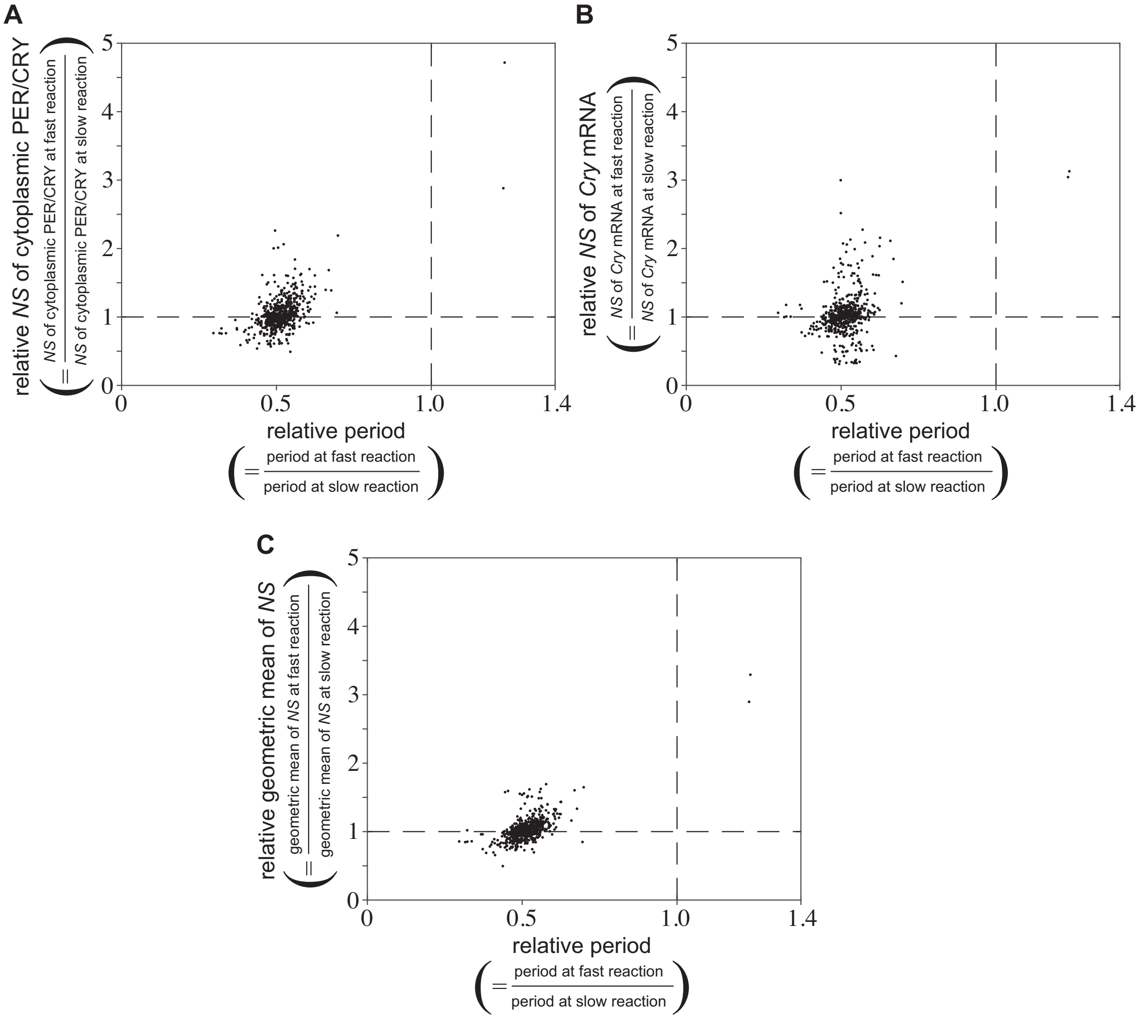

Figure S 5: Distribution of the relative NS of the Relógio model [73] as a function of the relative period when rate constants are increased by a factor of $1.5 - 2.5$. We plot relative NS of cytoplasmic PER/CRY complex (A), *Cry* mRNA (B), and geometric mean of NS for all variables (C).
