# Supplementary material for: Waveform distortion for temperature compensation and synchronization in circadian rhythms: An approach based on the renormalization group method
Source: PLoS Comput Biol. 2025 Jul 22;21(7):e1013246. doi: 10.1371/journal.pcbi.1013246 (PMC12282898; doi:10.1371/journal.pcbi.1013246)
Supplement: S6 Fig — We first generated reference parameter sets. Then, each parameter a, b, ε, and ε′ in the model’s reference parameter set was randomly multiplied by a factor of 1.1-1.9. (C) Examples of the waveform in the Lotka-Volterra model when the period is relatively unchanged. The blue and red lines represent small and large parameters, respectively. (PDF) [file pcbi.1013246.s011.pdf]

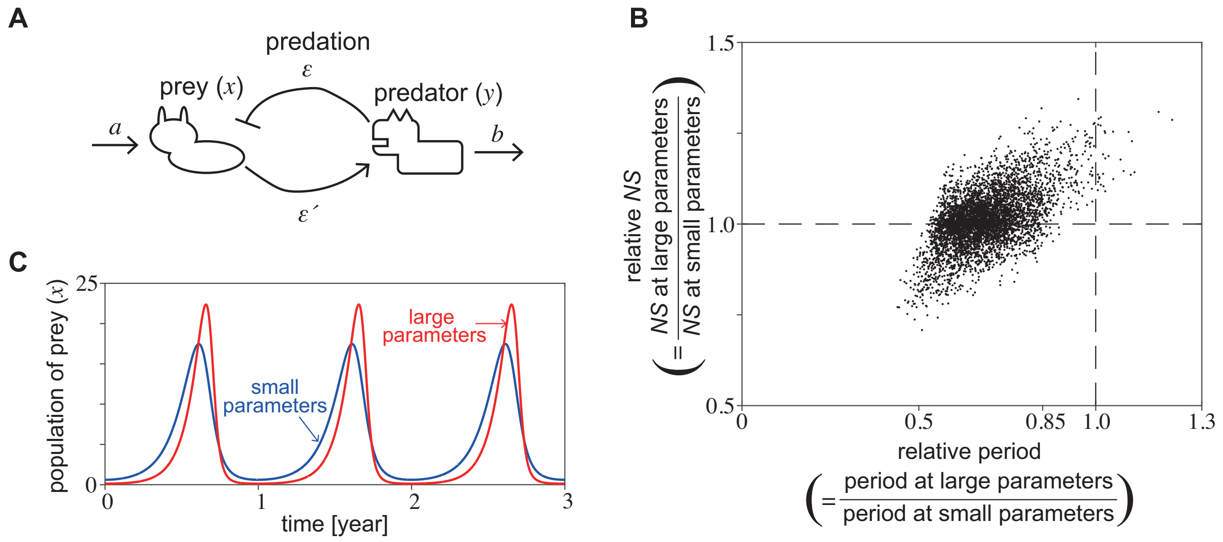

Figure S 6: (A) Lotka-Volterra model. (B) Distribution of relative NS for $x(t)$ of the Lotka-Volterra model as a function of the relative period when rate constants are increased. We first generated reference parameter sets. Then, each parameter a, b, ε, and ε' in the model's reference parameter set was randomly multiplied by a factor of 1.1-1.9. (C) Examples of the waveform in the Lotka-Volterra model when the period is relatively unchanged. The blue and red lines represent small and large parameters, respectively.
