# Supplementary material for: Waveform distortion for temperature compensation and synchronization in circadian rhythms: An approach based on the renormalization group method
Source: PLoS Comput Biol. 2025 Jul 22;21(7):e1013246. doi: 10.1371/journal.pcbi.1013246 (PMC12282898; doi:10.1371/journal.pcbi.1013246)
Supplement: S7 Fig — The parameter value is ε=0.1 (blue) and ε=3 (red). (PDF) [file pcbi.1013246.s012.pdf]

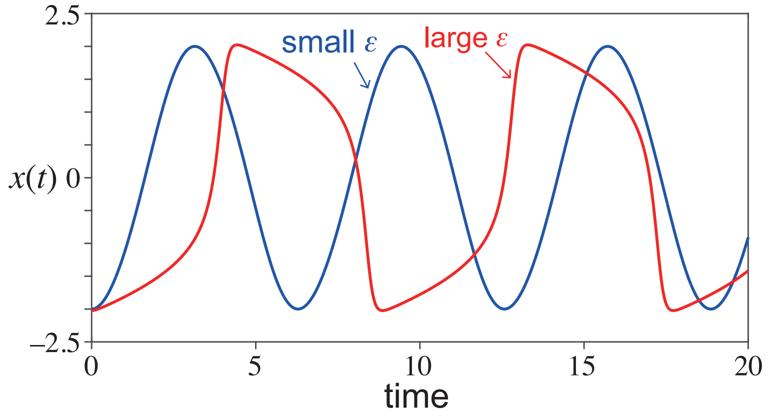

Figure S 7: The waveform examples of the van der Pol model. The parameter value is $\varepsilon = 0.1$ (blue) and $\varepsilon = 3$ (red).
