# Supplementary material for: Waveform distortion for temperature compensation and synchronization in circadian rhythms: An approach based on the renormalization group method
Source: PLoS Comput Biol. 2025 Jul 22;21(7):e1013246. doi: 10.1371/journal.pcbi.1013246 (PMC12282898; doi:10.1371/journal.pcbi.1013246)
Supplement: S1 Table — (PDF) [file pcbi.1013246.s013.pdf]

Supplementary Tables

Table S 1: Activation energies and frequency factors for each reaction in Fig. 1

Parameter	Activation Energy, E_i	Frequency Factor, A_i
k_1	1.39×10^4	43.0
k_2	6.31×10^3	2.48
k_3	6.56×10^3	2.49
p_1	1.94×10^4	246
p_2	7.03×10^3	2.62
r	8.11×10^4	1.13×10^{13}
