# Supplementary material for: Waveform distortion for temperature compensation and synchronization in circadian rhythms: An approach based on the renormalization group method
Source: PLoS Comput Biol. 2025 Jul 22;21(7):e1013246. doi: 10.1371/journal.pcbi.1013246 (PMC12282898; doi:10.1371/journal.pcbi.1013246)
Supplement: S2 Table — (PDF) [file pcbi.1013246.s014.pdf]

Table S 2: Parameter values for each reaction in Fig. 3B-C and Fig. 6.

	Fig. 3B and Fig. 6		Fig. 3C	
Parameter	slow	fast	slow	fast
k_1	0.269	0.296	0.247	0.278
k_2	0.200	0.221	0.192	0.213
k_3	0.0817	0.150	0.0662	0.112
p_1	0.290	0.328	0.160	0.219
p_2	0.246	0.376	0.256	0.312
r	0.180	0.226	0.244	0.314
n	15	15	13	13
