# Supplementary material for: Waveform distortion for temperature compensation and synchronization in circadian rhythms: An approach based on the renormalization group method
Source: PLoS Comput Biol. 2025 Jul 22;21(7):e1013246. doi: 10.1371/journal.pcbi.1013246 (PMC12282898; doi:10.1371/journal.pcbi.1013246)
Supplement: Attachment — Submitted filename: Review_Aurore_Woller.pdf [file pcbi.1013246.s015.pdf]

Review:

Using a minimal molecular clock model the authors show both theoretically and numerically that the waveform distorsion is necessary for temperature compensation. The analysis of experimental data is in agreement with these theoretical results. The study also shows that the entrainment range decreases when temperature increases.This is an interesting work and I find the use of the renormalization group method particularly elegant. Here are a few questions and a few suggestions to improve the manuscript:

General comment:

The authors might consider re-organizing the structure of sub-sections 2 and 3 of the result section. Personnally I have had to go back and forth several times to understand what was done.

Specific comments:

L 135-137: the waveform distorsion (NS) shown on Figure 1B is really close to 1; it would be more convincing to show a case where the NS gets a bit larger.

L 151-153: I would suggest showing a figure with the amplitude as a function of the temparature.

L 205-206: It might be relevant to show an inset focusing on the area with temperature compensation ($0.85 <$ relative period < 1.15).

L 179-206: How much do these results extend to a more realistic mammalian clock model ? I would suggest testing this with the widely-used model of Relogio et al. (2011, Plos Comp)

L 239 - 240: How does A1 change when the reaction rates increase ?

L 266-292: It would be interesting to do the same analysis with mammalian data if possible.

L 280-283: I think a statistical test comparing the medians is missing in Figure 5B.

L 351-356: Does this have implications on how circadian clocks (and especially their entrainment) are affected by seasonal temperature changes ? Could authors discuss this point ?

Minor comments:

L 70: I wouldn't use the term "unresolved" issue.

L 295: there is a little typo "reslut"
