# Supplementary material for: Waveform distortion for temperature compensation and synchronization in circadian rhythms: An approach based on the renormalization group method
Source: PLoS Comput Biol. 2025 Jul 22;21(7):e1013246. doi: 10.1371/journal.pcbi.1013246 (PMC12282898; doi:10.1371/journal.pcbi.1013246)
Supplement: Attachment — Submitted filename: response_to_reviewers.pdf [file pcbi.1013246.s016.pdf]

Feilim Mac Gabhann, PhD and Virginia Pitzer, PhD

Editor-in-Chief

PLOS Computational Biology

Dear Prof. Gabhann and Prof. Pitzer:

We appreciate the opportunity to revise our manuscript, titled " Waveform distortion for temperature compensation and synchronization in circadian rhythms: An approach based on the renormalization group method " (Manuscript ID: PCOMPBIOL-D-24-01483). We are grateful to you and the reviewers for your critical reading of on our previous manuscript and your constructive comments. In this letter, we have provided point-by-point responses to the reviewers' comments. We hope that these revisions will meet the standards for publication in PLOS Computational Biology.

We thank you for your time and consideration.

Best regards,

Shingo Gibo, PhD, Senior Researcher, IBS, South Korea

Gen Kurosawa, PhD, Senior Research Scientist, RIKEN, Japan

The point-by-point responses to the comments

Comments from the reviewers are indicated by blue letters.

Reviewer 1:

This manuscript is an interesting follow-up of Ref.31 showing that the occurrence of temperature-compensation in biochemical oscillators is related to certain asymmetric concentration-time profiles. Here, the authors apply a renormalization method to show this. I have only a few comments, which I feel the authors should consider.

The suggestion that asymmetric concentration-time profiles appear necessary to get temperature-compensation seems to imply that when concentration profiles are completely symmetric, such as in feedbacks showing harmonic oscillations, then it should be difficult to introduce temperature compensation into these systems. Indeed, when analyzing a feedback mechanism that shows purely sinusoidal (i.e. harmonic) oscillations (see Fig S9 in Thorsen et al. PLoS ONE 9(9): e107766. doi:10.1371/journal.pone.0107766) this oscillator cannot be temperature compensated, because the frequency w there will depend only on the two rate parameters, k3 and VmaxEset, which both increase w according to $w^2 = k3$ .VmaxEset. I feel that this could be used in this ms. as an additional indicator to the suggested rule addressed here.

We appreciate the reviewer's positive comment on our main results. Thank you also for the suggestion. We agree that the findings of Thorsen et al. (2014), which showed that a simple oscillator model with sinusoidal oscillations cannot achieve temperature compensation, are important and indeed related to our current results. To emphasize this point, we have added the following sentences in Line 162.

"This result, based on the Goodwin model (Gibo and Kurosawa (2019)), which demonstrated that a more distorted waveform at higher temperatures is both necessary and sufficient for temperature compensation, aligns with a previous study (Thorsen et al (2014)) showing that an alternative oscillator model with sinusoidal oscillations alone cannot achieve temperature compensation."

Concerning the renormalization method: By going through the supporting material this method

is quite theoretical/technical and requires some knowledge in perturbation theory. I think it would be good if the authors could include a section in the manuscript describing in ordinary terms the essence of this method with respect to oscillations for the less mathematically inclined reader (but still familiar with computations).

Thank you for the suggestion. To help non-specialists better understand the renormalization group method, we have included a brief introduction along with a simple illustrative example in the newly added Section 3.2. Although the section may appear somewhat long, we believe it is necessary to concisely explain the overall process of the renormalization group method.

Concerning Eq. 5: Although the authors refer to Forger's paper, it would improve the ms. to briefly recapitulate the meaning of the aj's and the method Forger has applied to obtain this equation. From the data by Thorsen et al (referred above) a similar analysis could probably be conducted on the expressions of the derived frequencies/periods there for various negative feedback oscillators, which included also a version of the Goodwin oscillator.

Thank you for raising these important points. We agree that adding an explanation of the meaning of a_j, the Fourier coefficients, as well as a brief outline of the derivation of the period formula, is helpful for the reader. We also appreciate you pointing out that a similar analysis for deriving the period of another oscillator was conducted in Thorsen et al (2014). To clarify and emphasize these points, we have added the following sentences in Line 149.

"Forger derived the period of Goodwin model with linear degradation (2)-(4) by applying signal processing methods (Forger (2011)), while a similar analysis has also been conducted for other negative feedback oscillators (Thorsen et al (2014)). First, Eqs. (2)-(4) were transformed into a single equation with higher-order derivatives (see Eq. (47) in S2 Text.). Next, the nonlinear term $f(x_3)$ was eliminated by multiplying the transformed equation by dx_3/dt and integrating for one period. Then, by expressing the periodic solution in Fourier series as $x_3(t) = \sum_{j=-\infty}^{\infty} a_j \exp(i(\frac{2\pi}{\tau})jt))$, the period was derived as follows:

$$\tau = \frac{2\pi}{\sqrt{k_1k_2 + k_2k_3 + k_3k_1}} \left[\frac{\sum_{j=1}^{\infty} |a_j|^2 j^4}{\sum_{j=1}^{\infty} |a_j|^2 j^2} \right]^{\frac{1}{2}}, \tag{1}$$

where a_j is the Fourier coefficient of $x_3(t)$. If $x_3(t)$ is distorted from a sinusoidal wave, the higher-order components a_j ($j \geq 2$) become large, causing NS to be large."

Line 54: The authors write: " One unresolved issue···.. " and refer to several suggestions how temperature-compensation could be understood. I suggest to rephrase this sentence in the sense that these different approaches do not necessarily exclude each other and represent different aspects of how to approach/describe temperature-compensation. In his book " The Geometry of Biological Time" Art Winfree has a nice compilation of different suggested mechanisms for temperature-compensation (some not mentioned by the authors) to which the authors could/should refer to.

Thank you for the clarification. We agree that the proposed mechanisms for temperature compensation are not mutually exclusive, as we stated in Line 139 of the previous version of the manuscript. To clarify this point, we have added the following sentences in Line 68.

"These proposed mechanisms, along with other mechanisms discussed in Winfree (2001), are not necessarily mutually exclusive and may work in combination to achieve temperature compensation."

Also note that there is an earlier paper on the temperature-compensated Goodwin oscillator (JTB (1996) 179:275ff) describing many circadian clock properties, including temperature entrainment, phase resetting by temperature steps, etc, which the authors may refer to (since in this paper the authors focus on the temperature-compensated Goodwin oscillator).

Thank you for bringing this important seminal study to our attention. We have now cited Ruoff and Rensing (1996) in a newly added sentence in Line 406.

"To investigate these conditions, we focus on the Goodwin model, which is widely used as a mathematical model that simulates various properties of biological clocks, including temperature compensation, synchronization to temperature cycles, and phase resetting by temperature steps (Ruoff and Rensing (1996))."

Be aware that Goodwin formulated the oscillator using two versions: (i) one where removal reactions are zero-order with respect to the degraded species and where an inhibitory cooperativity of one is sufficient to obtain oscillations, and (ii) a second formulation where removal reactions are first-order with respect to the degraded species and which require a much higher cooperativity of the inhibitor. This has been summarized in a recent review by in Biotheoretica 2020, doi: 10.1007/s10441-020-09379-8, which may also be of interest to the reader with respect to the Goodwin oscillator.

Thank you for the comment. We agree that statement that Goodwin formulated the oscillator using two versions can be of interest to the reader. To stress this point, we have added the explanation for the Goodwin model in Line 144:

"In fact, there are two versions of the Goodwin model: (i) one in which the degradation rate is expressed as a linear function of the substrate, as in Eqs. (2)–(4), and which requires high cooperativity (n) for oscillations; and (ii) another formulation in which the degradation rate follows a Michaelis-Menten equation, where a cooperativity of one is sufficient to generate oscillations (Goldbeter (1995); Kurosawa and Iwasa (2002); Gonze and Ruoff (2021)). In the present paper, we assume that the degradation rate follows a linear function of the substrate, for the sake of simplicity and analytical tractability."

Finally, concerning the verification of the model with respect of experimental data on page 13: the authors could further make connection to a paper from 1997 (Chronobiology International 14: 499-510, Fig. 3) which describes the temperature behavior of the per and frq mutants in Drosophila and Neurospora by the Goodwin oscillator.

Thank you for the constructive suggestion. We agree that the predictions of our model can be tested using other experimental data. In this revision, following your advice as well as that of Reviewer 3, we have analyzed mammalian experimental data from Zhou et al. (2015) and confirmed the prediction. We also agree that the prediction can be further tested using data from per and frq mutants in Drosophila and Neurospora, as you suggested. To emphasize these points, we have added the following sentences in Line 375:

"To examine the generality of the results, we also tested our conclusion using mouse embryonic fibroblasts (MEFs) data (Zhou et al. (2015)). According to our prediction, the waveform of the circadian gene activity (Per2-luc) in wildtype mice should become more non-sinusoidal at higher temperatures. Indeed, the NS value was significantly higher at elevated temperatures ($t = 2.9793$, $df = 189$, $p = 0.0016$), supporting the generality of our findings (S4 Fig.)."

We have also added the following sentence in the Discussion section (Line 470):

"Our theoretical prediction can be tested in circadian organisms such as mice and Neurospora (Ruoff 1997)."

There are also ultradian rhythms which show temperature-compensation. I feel the authors should extend their discussion briefly also in this direction.

Thank you for the comment about ultradian rhythms with temperature compensation. We have added a sentence in Line 55:

"Temperature compensation of the period occurs not only in circadian rhythms but also in ultradian rhythms, such as yeast metabolic cycles (Chen (2007); Kurosawa et al (2017))."

Reviewer 2:

The authors address a problem of general interest – the experimentally observed temperature compensation of circadian clock and the entrainment range of circadian rhythms by external zeitgebers.

Figure 1 summarizes the main findings based on numerical simulations of a specific model. The main achievements of the manuscript are the generalization of the findings regarding higher harmonics in the biological context (theories II, II, and III) and the generic discussion of the results using the renormalization group method.

I find the combination of extensive simulations (Figure 2), semi-analytical calculations (Figure 4), and experimental data (Figure 5) convincing. Other popular models (Lotka-Volterra, van der Pol, FitzHugh-Nagumo) confirm the main results.

Specific comments: Line 27: Renormalization group theory was applied also earlier to biological problems. An example is the Feigenbaum scenario in iterated maps from population dynamics.

We appreciate the reviewer's positive comment on our main results. Thank you also for raising an important point, stating: " Renormalization group theory was applied also earlier to biological problems. An example is the Feigenbaum scenario in iterated maps from population dynamics. "

To our knowledge, however, there are different approaches within renormalization group theory, and the approach used in the Feigenbaum scenario from iterated maps in population dynamics, while related, differs from the approach adopted in our analysis. To clarify this point, we have added the following explanation in the main text at Line 89.

"As elucidated in a historical review by Shirkov (1998), there are several distinct approaches that have been referred to collectively as the renormalization group (RG) method. Among these approaches, concepts such as asymptotic functional self-similarity, reminiscent of RG techniques used in extracting critical exponents in statistical physics (Castro and Jona-Lasinio, 1969; Wilson and Kogut, 1974; Wegner and Houghton, 1973), were notably applied by Feigenbaum (1979) to derive universal constants characterizing bifurcation points in certain iterative maps encountered in population biology (May, 1976; May and Oster, 1976). In contrast, the RG method utilized in this current work (Chen et al., 1994; Kunihiro, 1995; Veysey and Goldenfeld, 2007) is an adaptation of the approach originally developed in quantum field theory (Gell-Mann and Low, 1954; Bogoliubov and Shirkov, 1959). This approach provides a straightforward resummation technique for perturbative solutions, thus offering practical approximate solutions valid over global time domain."

Line 35: Narrowing of the entrainment range is an important finding. However, also large amplitudes of strong oscillators lead to narrow entrainment ranges (see e.g. Aschoff and Pohl 1978). Is NS related to increasing amplitudes? Moreover, amplitude-period correlations termed " twist " lead to a skewing and possibly to a narrowing of Arnold tongues.

Thank you for the positive comment and for raising this important issue. In our previous pa-

per (Gibo and Kurosawa, 2019, Biophysical Journal), we showed that both amplitude and NS increase with temperature in a realistic mammalian model, under the assumption that all reactions accelerate with temperature and the period is temperature-compensated. In the present revision, we have numerically demonstrated that amplitude also increases with temperature in the Goodwin model under temperature-compensated conditions, suggesting that NS is related to increasing amplitudes, and that a more distorted waveform (i.e., a larger NS) leads to a narrower entrainment range. To illustrate these findings, we have added the corresponding sentences in Lines 450.

"To compare the role of waveform with that of amplitude in temperature compensation and synchronization, we also analyzed amplitude. Specifically, we numerically showed that the amplitude also becomes larger at higher temperatures when the period is stable to changes in temperatures (S1 Fig.), which is consistent with previous study (Lakin-Thomas et al. (1991); Kurosawa et al. (2017)). The correlation between period and amplitude is called "twist" (Olmo et al. (2023)). It has been known that large amplitudes lead to narrow synchronization region (Abraham et al. (2010); Aschoff and Pohl (1978)). These might indicate that both the large amplitudes and distorted waveform lead to similarly narrow synchronization region."

Line 89: I find the long list of references unfocussed. Moreover, classical old papers are not cited (Bogolubov, Wilson, Feigenbaum).

Thank you for pointing out the missing reference and for your helpful comment regarding the clarity of citations. We agree that additional explanation is necessary when listing multiple references related to RG theory. To improve clarity, we now cite these references separately, according to the specific context in which each study was conducted. We have made this change in Line 87.

"To this end, we employ the renormalization group (RG) method [41, 42, 43, 44, 45, 46] adapted for global and asymptotic analysis of differential equations on the basis of the perturbation theory [47, 48, 49, 50, 51, 52, 53]. As elucidated in a historical review by Shirkov [54], there are several distinct approaches that have been referred to collectively as the renormalization group (RG) method. Among these approaches, concepts such as asymptotic functional self-similarity,

reminiscent of RG techniques used in extracting critical exponents in statistical physics [44, 45, 46], were notably applied by Feigenbaum [55] to derive universal constants characterizing bifurcation points in certain iterative maps encountered in population biology [56, 57]. In contrast, the RG method utilized in this current work [47, 48, 51] is an adaptation of the approach originally developed in quantum field theory [42, 43]. This approach provides a straightforward resummation technique for perturbative solutions, thus offering practical approximate solutions valid over global time domain. The method also serves as a powerful reduction theory of the dynamics in the asymptotic region. The method has been applied to various models, including ordinary and partial differential equations, discrete maps, and stochastic equations [48, 49, 50, 52, 53, 58]. The origin of the powerfulness of the RG method as a tool of the global analysis can be intuitively understood by reformulating the method in terms of the classical theory of envelopes [48, 53]: The envelope of a set of perturbative solutions which are only valid only locally around the arbitrary initial time t_0 can be set up to constitute a valid solution to the equation in a global domain."

Line 87-103: We have added the following classical old papers on the renormalization group method:

[41] Ernst Carl Gerlach Stueckelberg and Andreas Petermann. Normalization of constants in the quanta theory. Helv. Phys. Acta, 26:499-520, 1953.

[42] Murray Gell-Mann and F. E. Low. Quantum electrodynamics at small distances. Phys. Rev., 95:1300-1312, 1954.

[43] N. N. Bogoliubov and D. V. Shirkov. Introduction to the Theory of Quantized Fields. Wiley-Interscience, N.Y., 1959.

[44] Carlo Di Castro and Givanni Jona-Lasinio. On the microscopic foundation of scaling laws. Phsyics Letters A, 29(6):322-323, 1969.

[45] K. G. Wilson and John B. Kogut. The Renormalization group and epsilon expansion. Phys. Rept., 12:75-199, 1974.

[46] Franz J. Wegner and Anthony Houghton. Renormalization group equation for critical phenomena. Phys. Rev. A, 8:401-412, 1973.

[54] D. V. Shirkov. The Bogoliubov renormalization group in theoretical and mathematical

physics. In Ringberg Symposium on Quantum Field Theory (On the Occasion of Wolfhart Zimmermann's 70th Birthday), 1998.

[55] Mitchell J Feigenbaum. The universal metric properties of nonlinear transformations. Journal of Statistical Physics, 21:669-706, 1979.

Line 209: Is " always " correct? I see some exceptions in Figure 2.

Thank you for raising this important point. In this manuscript, we theoretically and numerically show that NS tends to be larger at higher temperatures in the Goodwin model when the period is maintained under the assumption that reaction rates increase with temperature. As the reviewer pointed out, there are a few exceptions in Figure 2 where the period remains relatively stable (i.e., relative period is around 0.85–0.9) without a increase in NS. These cases occur only when the increase in reaction rates is minimal (e.g., by a factor of ~ 1.1), which is biochemically unrealistic. In most biological systems, reaction rates typically double or triple with a 10 $^\circ$ C increase in temperature (Rayes, Pendergast, and Yamazaki, 2008, Journal of Biological Rhythms). To reflect this nuance more accurately, we have removed the word " always " from Line 284 in this revision.

" In the previous section, we demonstrated that the temperature compensation of the period in the Goodwin model is accompanied by an increase of the index NS and waveform distortion correspondingly occurs as temperatures increase. "

Line 272: There is some redundancy in Results and Methods (e.g. WebPlotDigitizer etc.).

Thank you for the suggestion. To avoid redundancy, we have revised the explanation of the analysis of the experimental data in the Results section (Line 348) as follows:

"First, we extract the time series of the average curve from Fig. 3C in a prior study (Kidd et al. 2015). Then, we add uniformly distributed noise between -0.4 and 0.4 to the extracted data to consider data errors (Fig. 5A). The Fourier coefficients are estimated using generalized harmonic analysis (GHA) (see Methods for details)."

Reviewer 3:

Using a minimal molecular clock model the authors show both theoretically and numerically that the waveform distorsion is necessary for temperature compensation. The analysis of experimental data is in agreement with these theoretical results. The study also shows that the entrainment range decreases when temperature increases.This is an interesting work and I find the use of the renormalization group method particularly elegant. Here are a few questions and a few suggestions to improve the manuscript:

General comment:
The authors might consider re-organizing the structure of sub-sections 2 and 3 of the result section. Personnally I have had to go back and forth several times to understand what was done.

We appreciate the reviewer's positive feedback on our main results, as well as the insightful comment regarding the manuscript's structure. While we prefer to maintain the current structure, we agree that additional clarification would benefit readers. Therefore, we have included a more detailed explanation in the Introduction to better guide readers through the manuscript. Specifically, we have added the following sentences at Line 117.

"This paper is organized as follows. In Section 2, we introduce the index for waveform distortion and the Goodwin model, and summarize the main results regarding temperature compensation and synchronization of circadian rhythms. In Section 3, we investigate the conditions for temperature compensation. We present our results based on the renormalization group (RG) method in Subsections 3.1, 3.2 and 3.3, followed by experimental verification of the model's predictions in Subsection 3.4. In Section 4, we examine the conditions for synchronization. Section 5, the final section, is devoted to discussions. Some detailed technical account of our method of analysis and numerical analyses are given in Supporting Information."

Specific comments:
L 135-137: the waveform distorsion (NS) shown on Figure 1B is really close to 1; it would be more convincing to show a case where the NS gets a bit larger.

Thank you for the valuable suggestion. In response to this comment, we have numerically searched for a parameter condition in which the increase in NS due to temperature change is more prominent. We have replaced Figure 1B with a new version based on this updated calculation, using the parameter set shown in S1 Table.

L 151-153: I would suggest showing a figure with the amplitude as a function of the tempara-ture.

Thank you for the suggestion. In response to this comment, we have numerically analyzed the amplitude of circadian oscillations as a function of temperature when the period was temperature compensated. Based on this analysis, we have added S1 Fig. We also added the following sentence in Line 450.

"To compare the role of waveform with that of amplitude in temperature compensation and synchronization, we also analyzed amplitude. Specifically, we numerically showed that the amplitude also becomes larger at higher temperatures when the period is stable to changes in temperatures (S1 Fig.), which is consistent with previous study (Lakin-Thomas et al (1991); Kurosawa et al (2017))."

L 205-206: It might be relevant to show an inset focusing on the area with temperature compensation ($0.85 <$ relative period < 1.15).

Thank you for the suggestion. In response to this comment, we have inserted the temperature compensation range ($0.85 <$ relative period < 1.15) in Figure 2A. We believe this will help readers better understand the numerical results.

L 179-206: How much do these results extend to a more realistic mammalian clock model ? I would suggest testing this with the widely-used model of Relogio et al. (2011, Plos Comp)

Thank you for the important suggestion to extend our analysis to a more realistic mammalian clock model. In fact, we previously showed that NS becomes larger at higher temperatures

using a realistic mammalian clock model based on Zhou et al. (2015), as reported in Gibo and Kurosawa (2019). Following your advice, we have now numerically analyzed the Relógio model (2011). The NS values increased when the period remained unchanged or became slightly longer under the condition that rate constants were increased, consistent with our prediction. To illustrate these results, we have added a figure showing the correlation between the relative NS and the period in the Relógio model as S5 Fig., have added sentences in Line 415 and the detail explanation in S3 text.

"Notably, the relation between the period and waveform distortion holds not only for the Goodwin model but also for more realistic models. We have numerically showed the NS is larger when the period is stable to changes in temperature by using Zhou model, which includes 190 variables (Zhou et al. (2015); Gibo and Kurosawa (2019)). Additionally, we numerically analyzed the period and NS of Relógio model, which includes 19 variables (Relógio et al. (2011)), when all reaction rates increased by a factor of $1.5 - 2.5$ (see S3 text for details). When the period is stable or increases with increasing reaction rates, geometric mean of NS becomes larger (S5 Fig.). This suggests that the waveform distortion is important for temperature compensation in realistic circadian clock models."

We believe that this addition has improved our manuscript, and we thank the reviewer for the valuable suggestion.

L 239 - 240: How does A1 change when the reaction rates increase ?

Thank you for the insightful question. In the present revision, we have numerically analyzed how A_1 changes in response to increased reaction rates. Based on this analysis, we have added a new figure, S2 Fig., which shows the elasticity of A_1 ($= \partial \ln A_1 / \partial \ln q_i$, where q_i is k_1, k_2, k_3, p_1, p_2, and r). We have also added corresponding sentences in Line 300.

"For example, we analyzed how the value of A_1 in Eq. (20) depends on the reaction rates. This parameter is associated with the amplitude of the second harmonic component. To quantify its sensitivity to changes in the reaction rates, we used elasticity, defined as $\partial \ln A_1 / \partial \ln q_i$, where q_i represents the parameters k_1, k_2, k_3, p_1, p_2, and r. We found that A_1 increased or

decreased with increasing degradation rates (k_1, k_2, and k_3), and consistently decreased with increasing phosphorylation and transcription rates (p_1, p_2, and r). The sum of the elasticities, $\sum \partial \ln A_1/\partial \ln q_i$, was always zero, which reflects the fact that A_1 remains unchanged when all reaction rates are scaled by the same factor (S2 Fig.)."

L 266-292: It would be interesting to do the same analysis with mammalian data if possible.

Thank you for the valuable suggestion. In this revision, we analyzed mammalian *Per2-luc* time-series data from Fig. 5C of Zhou et al. (2015). We found that the NS value is larger at higher temperatures, consistent with our theoretical prediction. Based on this result, we have added a new figure (S4 Fig.) and corresponding sentences in Line 375.

"To examine the generality of the results, we also tested our conclusion using mouse embryonic fibroblasts (MEFs) data (Zhou et al (2015)). According to our prediction, the waveform of the circadian gene activity (Per2-luc) in wildtype mice should become more non-sinusoidal at higher temperatures. Indeed, the NS value was significantly higher at elevated temperatures (t = 2.9793, *df* = 189, $p = 0.0016$), supporting the generality of our findings (S4 Fig.."

L 280-283: I think a statistical test comparing the medians is missing in Figure 5B.

Thank you for the valuable suggestion. In this revision, we statistically compared the distributions of estimated NS values at different temperatures in the Drosophila experimental data using a t-test. We confirmed that the increase in estimated NS values with temperature was statistically significant in both the wild-type and the perL mutant, supporting our theoretical predictions. Based on these results, we have added corresponding explanations in Line 470.

"To be precise, we performed a t-test to compare the two groups at higher and lower temperatures. The difference in estimated NS values between the two temperature conditions was statistically significant in the wild-type ($t = 9.5755$, *df* = 193, $p < 0.001$) (Fig. 5). In the perL mutant, the difference was also statistically significant ($t = 14.7971$, *df* = 191, $p < 0.001$) (S3 Fig.), supporting our theoretical predictions."

L 351-356: Does this have implications on how circadian clocks (and especially their entrainment) are affected by seasonal temperature changes ? Could authors discuss this point ?

Thank you for the interesting question. We do not yet know the precise implications of how the entrainment of the circadian clock is affected by seasonal changes in temperature. In this manuscript, we analytically and numerically show that synchronization with light-dark cycles becomes more difficult at higher temperatures. These results are consistent with previous experimental findings in Drosophila and Neurospora, which showed that the magnitude of phase shifts in response to light pulses decreases at higher temperatures (Nakashima and Feldman (1980); Varma et al (2013)).

These findings may offer some hints about how seasonal temperature changes affect circadian entrainment. However, further studies are needed, as light intensity also varies seasonally. To clarify this point, we have added the following sentences in Line 446:

”These results suggest that the waveform of gene and/or protein activity may vary seasonally between hot summers and cold winters, potentially affecting the entrainment of circadian clocks. Further studies are needed to investigate this possibility, particularly in light of seasonal variations in both temperature and light intensity.”

Minor comments:

L 70: I wouldn't use the term “ unresolved ” issue.

We have revised the sentence in Line 71 as follows:

”Another key issue in circadian rhythm research is synchronization with 24-hour environmental light-dark cycles.”

We have also revised the sentence in Line 53 as follows:

”One fundamental issue that remains to be understood in circadian rhythm research is temperature compensation (Woodlan et al. (1957); Ruoff (1992); Ruby et al. (1999); Zhou et al.

(2015); Kurosawa et al. (2017); Sasai (2022); Schmal et al. (2023); Chakravarty et al. (2023)), in which the period keeps constant despite temperature-induced changes in reaction rates."

L 295: there is a little typo "reslut"

Corrected.
